# YOLO-DCPG: a lightweight architecture with dual-channel pooling gated attention for intensive small-target agricultural pest detection

**DOI:** 10.3389/fpls.2025.1716703

**Published:** 2025-12-11

**Authors:** Jingwei Liu, En Yu, Yongke Li, Yunjie Zhao, Bowen Mao

**Affiliations:** 1 College of Computer and Information Engineering, Xinjiang Agricultural University, Urumqi, China; 2 Xinjiang Agricultural Informatization Engineering Technology Research Center, Urumqi, China; 3 Research Center for Intelligent Agriculture, Ministry of Education Engineering, Urumqi, China

**Keywords:** agricultural pest detection, lightweight, DCPGAttention, StarNet, small-neck

## Abstract

Accurate and rapid identification of agricultural pests is essential for intelligent pest monitoring. However, existing pest detection models often suffer from high parameter counts and computational complexity, limiting their deployment on edge devices. To address these challenges, this paper proposes a lightweight agricultural pest detection model, YOLO-DCPG, based on YOLOv8n. First, a Dual Channel Pooling Gated Attention (DCPGAttention) module is designed, applying mean and standard deviation pooling to enhance global information capture. A lightweight backbone network, StarNet, is employed for feature extraction, while a feature fusion neck, Small-Neck, is introduced, based on an improved bidirectional feature pyramid network (a-BIFPN) and integrating the efficient GSConv module. This design reduces model parameters and computational cost while maintaining detection accuracy. Furthermore, a scale factor based on Inner-IoU is incorporated into the WIoU loss function, enabling more precise control over auxiliary bounding boxes and improving regression for small pest regions. Experimental results on the Pest24 dataset show that YOLO-DCPG achieves a precision of 80.1%, mAP@50 of 74%, and mAP@50~95 of 47.5%, representing improvements of 4.5%, 0.8%, and 0.9% over the baseline YOLOv8n, respectively. Meanwhile, the number of parameters, GFLOPs, and model size are reduced by 51.2%, 30.1%, and 46.7%, respectively. Finally, YOLO-DCPG is successfully deployed on Raspberry Pi 4B, achieving stable, real-time pest detection, demonstrating its effectiveness and practicality for edge agricultural applications.

## 1 Introduction

As one of the largest agricultural countries in the world, agricultural pests have seriously affected the growth of crops in China Sprague (1975). The wide variety of pest species and the difficulty in identifying them have long troubled agricultural practitioners. Traditional pest identification relies primarily on experts, which requires extensive professional knowledge and is time-consuming. Manual identification often fails to provide timely feedback on pest outbreaks, leading to missed opportunities for optimal pest control (Song et al., 2025). Therefore, timely and accurate pest detection is crucial for crop protection.

Traditional Automated pest identification methods mainly rely on image processing and feature engineering combined with machine learning algorithms for pest classification. Ebrahimi et al. (2017) employed Support Vector Machines (SVM) for automatic detection of thrips in strawberry greenhouses. Chodey and Shariff (2023) proposed a method for extracting texture features based on the Gray-Level Co-occurrence Matrix (GLCM). Amrani et al. (2024) proposed a pest detection method based on Bayesian multitask learning and conducted aphid detection experiments on images of various crops. The detection accuracies for aphids in corn, rapeseed, rice, and wheat images were 75.77%, 66.39%, 70.01%, and 59%, respectively. However, these methods were tested on images with simple backgrounds and only a few pest species. They did not fully consider the complexity of real agricultural environments and the diversity of pests. Although these methods show progress in pest detection and have simple structures with high computational efficiency, which is useful for real-time or resource-limited applications, they mainly rely on low-level features such as color and shape for classification. This limits the generalization ability of the models. In addition, most traditional methods focus on datasets with simple backgrounds and few pest types. This makes them less suitable for real-world conditions. Compared to them, deep learning methods use convolutional neural networks (CNNs) to extract global contextual features. This overcomes the limits of traditional methods and greatly improves the accuracy and robustness of agricultural pest detection.

In recent years, with the rapid development of convolutional neural networks (CNNs), agricultural pest detection has become a popular research area. Currently, CNN-based object detection algorithms are mainly divided into two-stage and single-stage methods.

The two-stage methods include Regions with Convolutional Neural Network features (R-CNN) (Girshick et al., 2014), Fast R-CNN (Girshick, 2015), and Faster R-CNN (Ren et al., 2016). These methods typically first generate candidate regions and then perform classification and bounding box regression for each region. For example, Ali et al. (2023)Ali et al. proposed a novel deep learning method called Faster-PestNet. It uses MobileNet to extract sample features and then employs an improved two-stage locator based on Faster R-CNN for pest recognition. The method achieved an accuracy of 82.43% on the IP102 pest dataset. However, it did not consider real agricultural environments or deployment on edge devices. Deepika et al. used Mask R-CNN to identify five types of pests and achieved good detection results. Nevertheless, their study focused on images with simple backgrounds and lacked research on insect detection in complex environments (Deepika and Arthi, 2022). Overall, two-stage methods have advantages in detection accuracy, but they typically incur high computational costs, making them less suitable for real-time applications and deployment on edge devices. The single-stage methods mainly include You Only Look Once (YOLO) (Adarsh et al., 2020; Bochkovskiy et al., 2020; Jocher et al., 2020; Li et al., 2022a; Khanam and Hussain, 2024; Varghese and Sambath, 2024; Wang et al., 2024) and Single Shot MultiBox Detector (SSD) (Liu et al., 2016). They are characterized by fast detection speed, making them suitable for real-time detection tasks. Depending on the research focus, they can be divided into small-pest detection methods and lightweight approaches.

In small-pest detection, certain studies have enhanced accuracy by incorporating feature enhancement modules and optimized loss functions. Dong et al. (2024) developed the PestLite model based on YOLOv5. By introducing a multi-level spatial pyramid pooling module (MTSPPF), the model effectively captures multi-scale features and achieved 90.7% mAP@50 on rice and corn pest detection tasks. Wen et al. (2022)Wen et al. proposed Pest-YOLO, a detection algorithm aimed at counting tiny pests. This method improves small target detection by incorporating an intersection loss function and a merging strategy. On the Pest24 dataset, Pest-YOLO achieved 77.71% recall and 69.59% mAP@50. However, the method only focuses on the number of detected pests and overlooks a comprehensive evaluation of detection accuracy, resulting in a low precision of 46.97%. Additionally, the model has a large number of parameters, which hinders deployment and real-time application on edge devices. Zhang et al. (2025) proposed an improved small-object detection model, FCDM-YOLOv8, for field crop pest detection. The model integrates lightweight modules, context feature enhancement, an additional small-object detection layer, and a dynamic detection head to improve accuracy and recall for dense and small pests. Experiments on VisDrone2019 and COCO2017-small demonstrated that FCDM-YOLOv8 outperforms YOLOv8n and other mainstream detectors. However, the model still shows limited mAP improvement and insufficient recall in complex scenarios. Liu et al. (2025) proposed an RP-DETR model for rice pest detection, which combines Transformers and CNNs. The model introduces a Small Target Improved Pyramid (STIP) in the neck, integrating Cross Stage Partial Networks and an Omni-Kernel Module with large convolution kernels to alleviate the problem of small-object feature loss and improve detection accuracy. However, the model suffers from high computational cost and relatively slow inference speed, which may limit its deployment in large-scale agricultural scenarios. Tang et al. (2023) proposed an enhanced version of Pest-YOLO by incorporating the ECA attention mechanism and a Transformer encoder to improve global feature capture. The model achieved 73.4% mAP@50 on the Pest24 dataset. However, this method did not adequately address model lightweight design and deployment on edge devices.

In terms of lightweight design, some studies have improved YOLO-series models through structural optimization and attention mechanisms. Dong et al. (2025b) proposed a lightweight deep learning model, REDNet, for real-time railway defect detection. By introducing efficient multi-scale feature aggregation and task decomposition modules, REDNet achieves high detection accuracy and fast inference speed, demonstrating the effectiveness of lightweight architectures in industrial inspection tasks. Similarly, Dong et al. (2025a) introduced RSNet, a lightweight and efficient detection framework tailored for small object detection in high-resolution remote sensing images. RSNet integrates compact detection heads and adaptive downsampling modules to enhance small-target sensitivity while maintaining computational efficiency, achieving state-of-the-art performance on DOTA and NWPU VHR-10 datasets. These studies highlight the versatility and effectiveness of lightweight architectures across diverse domains. In the agricultural field (Xue and Wang, 2025) proposed a lightweight detection model, PEW-YOLO. The model optimizes the PP-LCNet backbone with GSConv, introduces a lightweight PGNet backbone, integrates an efficient multi-scale attention mechanism in the neck, and adopts the Wise-IoU loss function. These improvements reduce parameters while enhancing accuracy and speed, enabling real-time detection of citrus pests and diseases. Huang et al. (2025) improved the YOLOv10n architecture using the SPD-Conv convolutional model, reverse residual attention mechanism, and Inner-SIoU loss function. Their model achieved 83.2% mAP@50 on the yellow sticky trap dataset with a computational complexity of 8.8 GFLOPs. Sun et al. (2025) proposed an improved storage pest detection algorithm based on YOLO11n, called PDA-YOLO, which primarily utilizes the PoolFormer_C3K2 module to reduce the model’s computational complexity to 6.9 GFLOPs. However, the dataset contains only a single pest species, resulting in limited generalization ability of the model. Yu et al. (2025) proposed a lightweight model, DGS-YOLOv7-Tiny, based on YOLOv7-Tiny and applied it to tomato pest detection. By introducing a global attention mechanism and a fused convolution structure, the model enhances small object detection capability while effectively reducing the number of parameters and computational complexity, thereby enabling efficient and real-time detection in edge computing environments.

Existing pest detection studies have achieved certain advances in accuracy, but they still share common limitations. First, most methods are trained on datasets with relatively simple backgrounds and limited pest species, resulting in poor generalization to complex real-world environments. Second, some approaches focus on improving small-object detection accuracy, which often leads to large model sizes and slow inference, hindering deployment on edge devices. Third, although lightweight methods reduce computational complexity, they often compromise detection accuracy across multiple pest species, making it difficult to achieve a balance between accuracy and model efficiency. To address this, this paper proposes a lightweight agricultural pest detection model named YOLO-DCPG based on YOLOv8n (Varghese and Sambath, 2024). The main idea is to optimize the model structure to reduce network parameters, computational complexity, and model size, while maintaining high detection accuracy. This enables better adaptation to edge device deployment. The main contributions of this work are as follows:

1. This paper proposes a DCPGAttention mechanism that uses mean and standard deviation pooling on input features to improve global information capture.
2. To balance detection accuracy and model lightweight design, the feature extraction backbone of YOLOv8n is replaced with the lighter StarNet. At the same time, a more efficient multi-scale feature fusion structure called Small-Neck is designed. This structure combines an improved a-BiFPN with the lightweight convolution module GSConv, which reduces computational cost while enhancing feature representation.
3. To accelerate model convergence, a scale factor based on Inner-IoU is introduced into the WIoU loss function to control auxiliary bounding boxes. This helps the model focus more on small pest regions and speeds up the regression of small targets, achieving a balance between detection accuracy and inference speed.
4. To further validate the practicality of the proposed lightweight model on edge devices, this paper deploys it on a Raspberry Pi 4B, achieving stable and real-time pest detection.

The structure of this paper is as follows: Section 2 mainly introduces the dataset acquisition and the YOLO-DCPG model architecture, with detailed descriptions of the improvements in each module. Section 3 presents the experimental design, result analysis, and edge device deployment. Section 4 provides the conclusion.

## 2 Materials and methods

### 2.1 Dataset introduction

**Figure 1:**
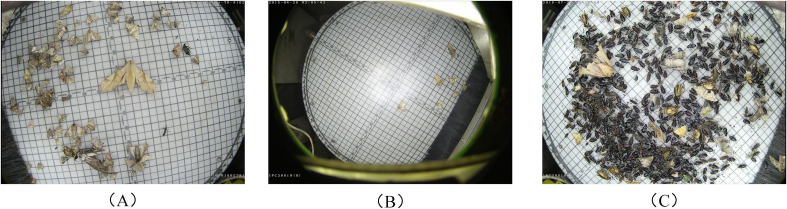
Sample images from the Pest24 dataset. **(A)** Images containing multiple pest types. **(B)** Samples captured at different scales. **(C)** Images showing dense pest distribution and occlusion.

The dataset used in this study is Pest24 (Wang et al., 2020a). It was collected by the Institute of Intelligent Machines, Chinese Academy of Sciences, using a self-developed pest image acquisition device. The dataset contains 25,378 images covering 24 distinct pest categories. Compared with general object detection datasets such as VOC and COCO, the targets in Pest24 are significantly smaller, and the images often contain complex scenes with overlapping and occluded objects. Some sample images are shown in **Figure 1** Additionally, Pest24 suffers from severe class imbalance, with large variations in the number of instances per category. For example, the 20th pest category (Anomala corpulenta) has 53,347 instances, while the 18th category (Holotrichia oblita) contains only 108. These characteristics make Pest24 a highly challenging dataset for object detection and place higher demands on model robustness and generalization.

To address the class imbalance problem, this paper adopts a combination of offline and online data augmentation methods. For offline augmentation, six techniques are applied: horizontal flipping, vertical flipping, random 90-degree rotation, brightness adjustment, horizontal translation, and vertical translation. These are used for pest categories with fewer instances, such as Land tiger, Eight-character tiger, Holotrichia oblita, and Nematode trench. For online augmentation, Mosaic (Bochkovskiy et al., 2020) and Mixup (Zhang et al., 2017) are employed during training to increase dataset diversity and improve model robustness. Mosaic augmentation combines four images into one, effectively enriching background textures and simulating multi-scale and occlusion conditions commonly observed in field pest detection scenarios. Mixup generates virtual samples by blending pairs of images and their corresponding labels, which helps the model learn smoother decision boundaries and reduces overfitting to specific pest categories. Incorporating spatial and illumination diversity through these augmentations improves the model’s ability to generalize across complex field conditions. As a result, the total number of images after augmentation reaches 29,680. The dataset is divided into training, validation, and test sets in a 7:2:1 ratio, with 20,776 images for training, 5,936 for validation, and 2,968 for testing.

### 2.2 YOLO-DCPG object detection model

YOLOv8 (You Only Look Once version 8), proposed by Ultralytics (Varghese and Sambath, 2024), consists of three main components: a backbone for feature extraction, a neck for feature fusion, and a detection head. The neck adopts a Path Aggregation Network (PAN) (Liu et al., 2018), which enhances the propagation of deep semantic features through a bottom-up information flow mechanism, thereby improving multi-scale feature fusion.

**Figure 2:**
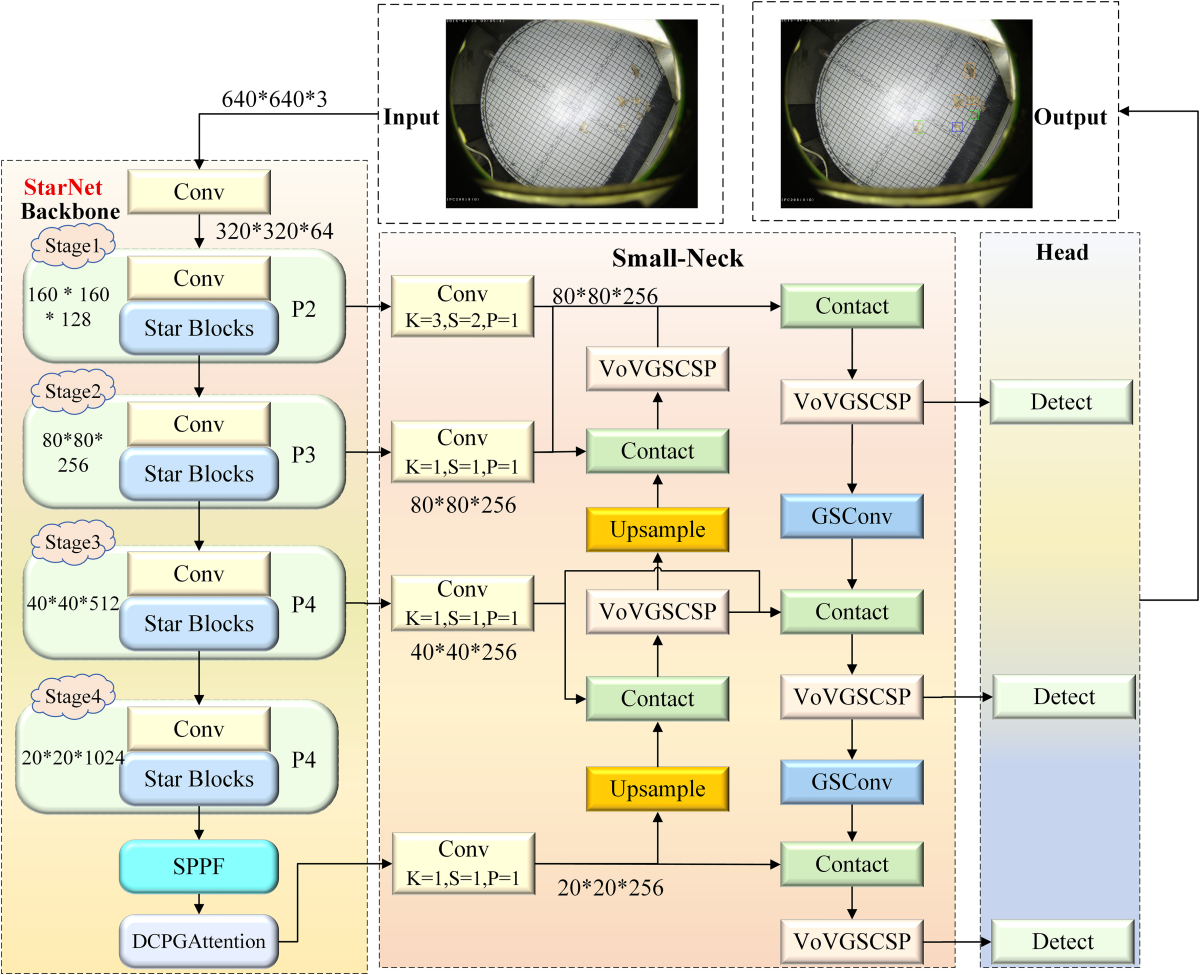
Architecture of the YOLO-DCPG model. The backbone employs StarNet-S100 for feature extraction, while the feature fusion network adopts a lightweight Small-Neck structure to enhance feature propagation and reduce parameters. The detection head retains the original YOLOv8 design for object classification and localization.

To address the deployment challenges of agricultural pest detection on edge devices caused by the large number of parameters in YOLOv8, we propose a lightweight model named YOLO-DCPG, which is built upon YOLOv8n and employs StarNet as the feature extraction backbone. The overall architecture of YOLO-DCPG is illustrated in **Figure 2**. First, a Dual Channel Pooling Gated Attention mechanism (DCPGAttention) is designed. It replaces the Global Context Embedding module of the Gated Channel Transformation (GCT) (Yang et al., 2020) attention mechanism with dual pooling operations based on mean and standard deviation. This module is integrated at the end of the backbone network to enhance the model’s ability to capture global pest information. Second, a lightweight Small-Neck network is designed to replace the neck of YOLOv8. It combines an enhanced bidirectional feature pyramid network (a-BiFPN) with the efficient convolution module GSConv (Li et al., 2022b), enabling effective fusion of features from different levels and better capturing multi-scale pest features. Additionally, in the loss function, a scale factor called ratio based on Inner-IoU (Zhang et al., 2023) is introduced into WIoU (Tong et al., 2023) to control the influence range of auxiliary bounding boxes. This helps the model focus more on small pest regions and accelerates the regression of small targets. The improved loss function enhances detection accuracy while effectively controlling computational complexity, achieving a balance between detection performance and inference efficiency. Through these three improvements, the model significantly reduces both parameter count and computational load.

#### 2.2.1 StarNet feature extraction network

**Figure 3:**
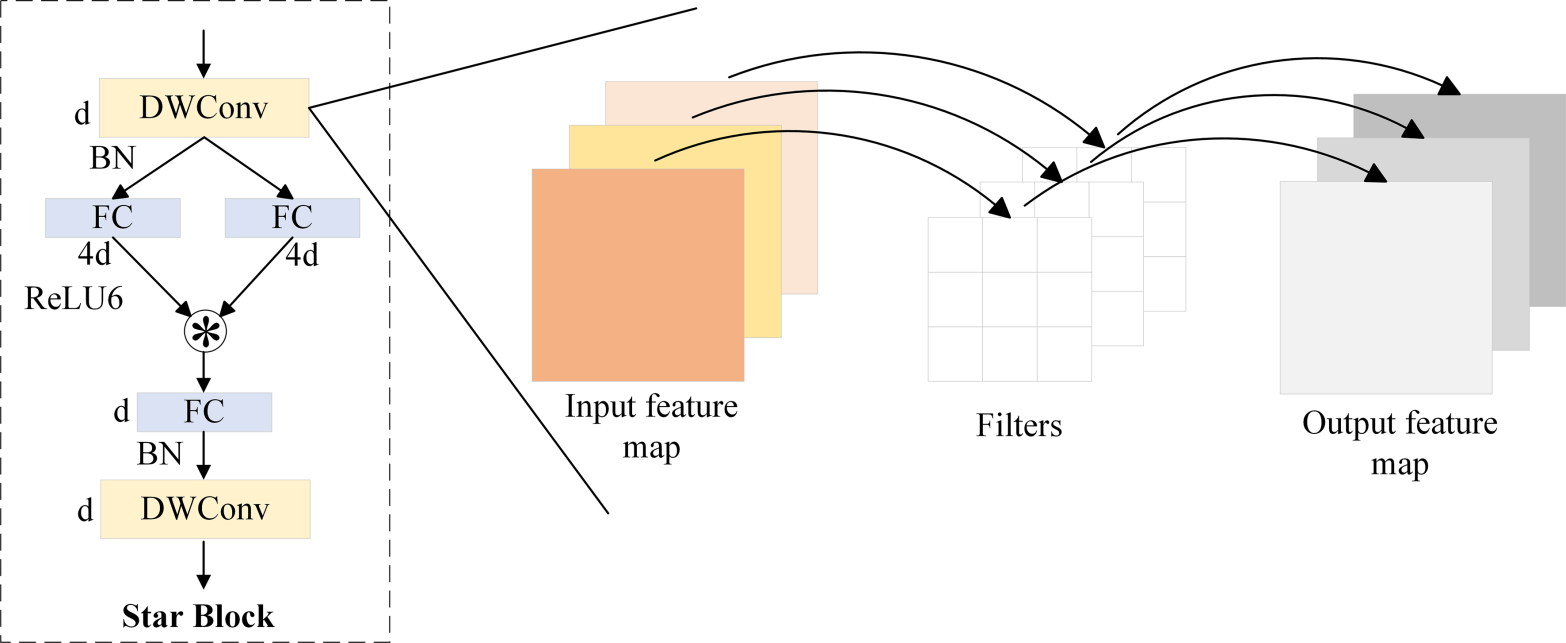
Structure of the Star Block. *denotes the star operation.

For pest detection tasks, high redundancy exists among feature maps, which affects model efficiency. Meanwhile, practical applications often rely on edge devices with limited computing resources. To reduce computational cost and parameter count, this paper adopts the lightweight and efficient StarNet-S100 (Ma et al., 2024) as the feature extraction backbone. It achieves a good balance between accuracy and computational overhead. The model structure is shown in the Backbone part of **Figure 2**. StarNet adopts a 4-stage hierarchical architecture, where downsampling is performed using convolutional layers. At each stage, a Star Block is introduced for feature extraction. The core component of the architecture is the Star Operation, an efficient feature transformation method based on element-wise multiplication, which enhances inter-channel modeling capability with extremely low computational cost. However, since the Star Operation inherently lacks spatial receptive fields, its ability to model local image structures such as edges and textures is limited. To address this issue, StarNet introduces Depthwise Convolution (DWConv) (Chollet, 2017) in each Star Block to enhance spatial modeling capability. In this design, the Star Operation is responsible for inter-channel interaction, while DWConv handles spatial feature extraction. The two components work together to achieve a balance between structural efficiency and representational capacity. In addition, to further improve detection efficiency and deployment performance, Batch Normalization is used after DWConv to replace the original Layer Normalization. This accelerates training convergence and simplifies computation. At the same time, a lightweight ReLU6 activation function is adopted to enhance non-linear modeling capability and reduce numerical instability. The structure of the Star Block is shown in **Figure 3**.

#### 2.2.2 DCPGAttention module

**Figure 4:**
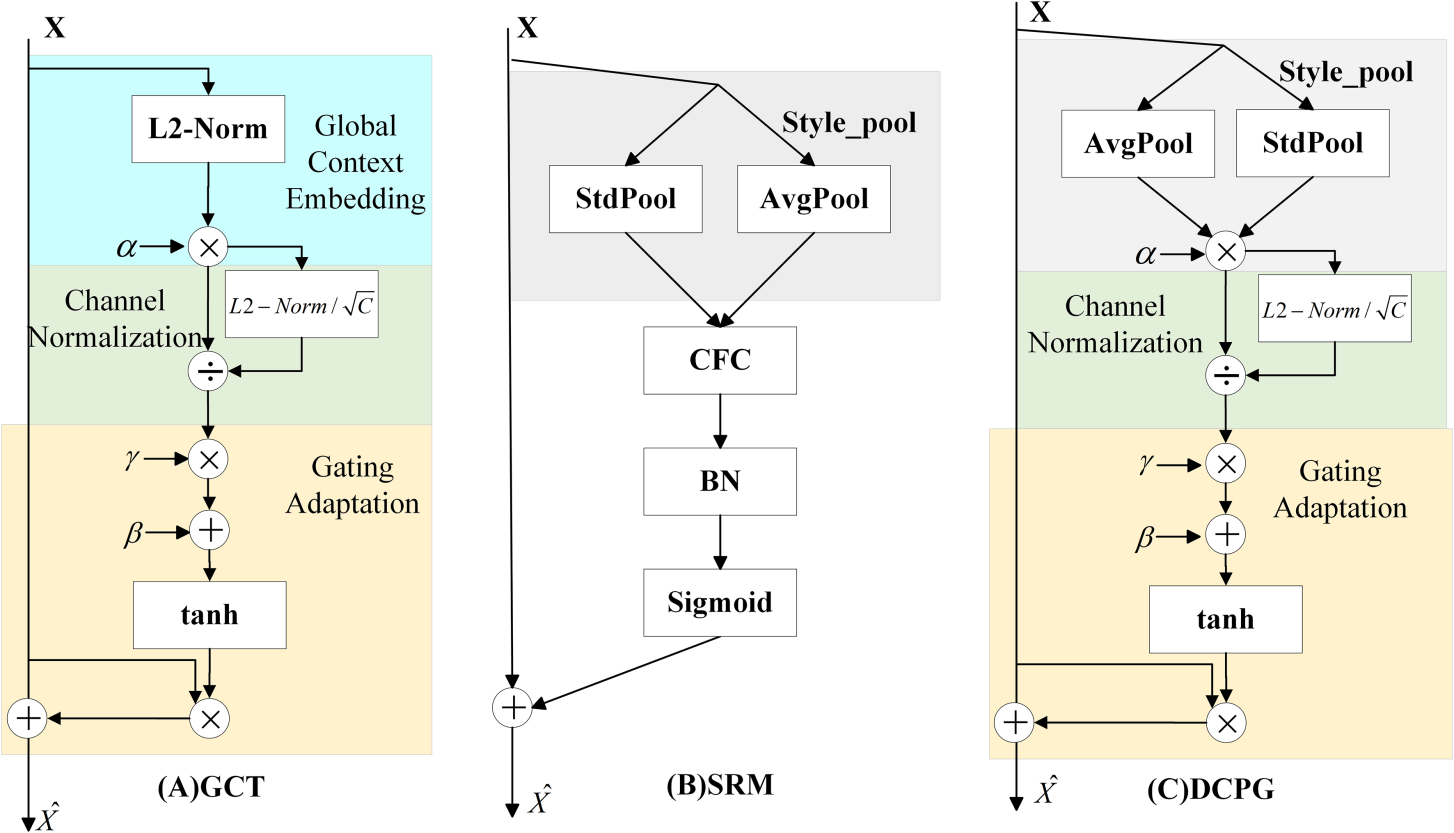
Diagram of the DCPG attention mechanism module.

The structure of the Gated Channel Transformation (GCT) (Yang et al., 2020) module is shown in **Figure 4A**. It consists of three parts: Global Context Embedding, Channel Normalization, and Gating Adaptation. Although the GCT module effectively introduces a learnable gating mechanism to adaptively adjust channel responses, its Global Context Embedding stage relies on L2-norm–based feature encoding, which enforces fixed mean values across channels. This design limits the ability to distinguish fine-grained feature variations and leads to the loss of informative distributional characteristics.

The Style-based Recalibration Module (SRM, **Figure 4B**) (Lee et al., 2019) aims to improve channel-wise feature representation by leveraging global statistical information. SRM extracts both the mean and standard deviation of each feature channel as style descriptors, capturing richer distributional characteristics compared to single-statistic methods. These dual statistics provide a more comprehensive global context representation and enable the network to recalibrate channel responses based on overall feature style information. However, the recalibration mechanism of SRM is fixed and input-independent, meaning that the weighting parameters remain constant once trained. As a result, SRM cannot flexibly respond to variations across different samples, which limits the model’s dynamic adaptability, generalization, and robustness in complex visual scenes.

To address these limitations, we propose the Dual Channel Pooling Gated Attention (DCPGAttention) module, as illustrated in **Figure 4C**. DCPGAttention integrates the dual-statistics pooling strategy of SRM with the adaptive gating concept of GCT to achieve both comprehensive and dynamically adjustable global feature modeling. Specifically, it applies mean and standard deviation pooling to extract richer contextual information, while a learnable weighting factor α adaptively balances the contribution of different channels. Compared with the L2-norm–based embedding in GCT, this design provides a more flexible and informative representation of global context. Given an input feature map $x\in R_{n\times c\times h\times w}$, the feature computation is defined as follows (**Equations 1**–**4**):

(1) $$\begin{array}{c} {\text{μ}}_{nc}=\frac{1}{HW}\sum_{h=1}^{H}\sum_{w=1}^{W}x_{c} \end{array}$$

(2) $$\begin{array}{c} {\text{σ}}_{nc}=\sqrt{\frac{1}{HW}\sum_{h=1}^{H}\sum_{w=1}^{W}{\left(x_{c}-{\text{μ}}_{nc}\right)}^{2}} \end{array}$$

(3) $$\begin{array}{c} t_{n}c=\left[{\text{μ}}_{nc},{\text{σ}}_{nc}\right] \end{array}$$

(4) $$\begin{array}{c} s_{c}={\text{α}}_{c}\left(t_{nc}+\text{ξ}\right) \end{array}$$

${\text{μ}}_{nc}$ denotes the mean of the channel features, and ${\text{σ}}_{nc}$ represents the standard deviation. $x_{c}$ is the input feature, where *H* and *W* are the spatial dimensions, *n* is the batch size, *c* is the number of channels, and *t* refers to the operation that integrates the dual pooled channel features. $\text{α}=\text{α}\left[{\text{α}}_{1},\cdots ,{\text{α}}_{c}\right]$ denotes the learned embedding weight, and ξ is a small constant added for numerical stability, preventing issues during backpropagation near zero.

In the Channel Normalization part, L2-norm is retained. This method normalizes the scale across channels while preserving the relative magnitude between channels. It introduces an attention mechanism that maintains higher activation values for strongly responding channels after normalization, thereby suppressing weakly responding channels and guiding the model to focus more on channels with prominent features. The normalization formula is defined as follows:

(5) $$\begin{array}{c} \hat{s_{c}}=\frac{\sqrt{C}s_{c}}{{\left[\left(\sum_{c=1}^{C}s_{c}^{2}\right)+\text{ξ}\right]}^{\frac{1}{2}}} \end{array}$$

In Equation 5, ξ is a small constant added for numerical stability to prevent division by zero. The scalar $\sqrt{C}$ is introduced to keep values within a reasonable range and avoid extreme magnitudes.

In the Gating Adaptation stage, the gating mechanism from GCT is retained. This mechanism introduces trainable parameters: a weight γ and a bias β, which control whether channel features are activated. A positive γ emphasizes inter-channel competition, while a negative γ promotes inter-channel cooperation. This dynamic mechanism allows the model to adaptively enhance or suppress channel features across different semantic levels, thereby improving its representational capacity. Gate control weight $\text{γ}=\left[{\text{γ}}_{1},\cdots ,{\text{γ}}_{c}\right]$, Gate control bias $\text{β}=\left[{\text{β}}_{1},\cdots ,{\text{β}}_{c}\right]$, The gate control function is formulated as **Equation 6**:

(6) $$\begin{array}{c} \hat{x_{c}}=x_{c}\left[1+\tanh\left({\text{γ}}_{c}{\hat{s}}_{c}+{\text{β}}_{c}\right)\right] \end{array}$$

In this equation, $x_{c}$ represents the original input channel feature. The final channel weight is represented by $1+\tanh\left({\text{γ}}_{c}{\hat{s}}_{c}+{\text{β}}_{c}\right)$.

#### 2.2.3 Small-neck: a lightweight feature fusion network

**Figure 5:**
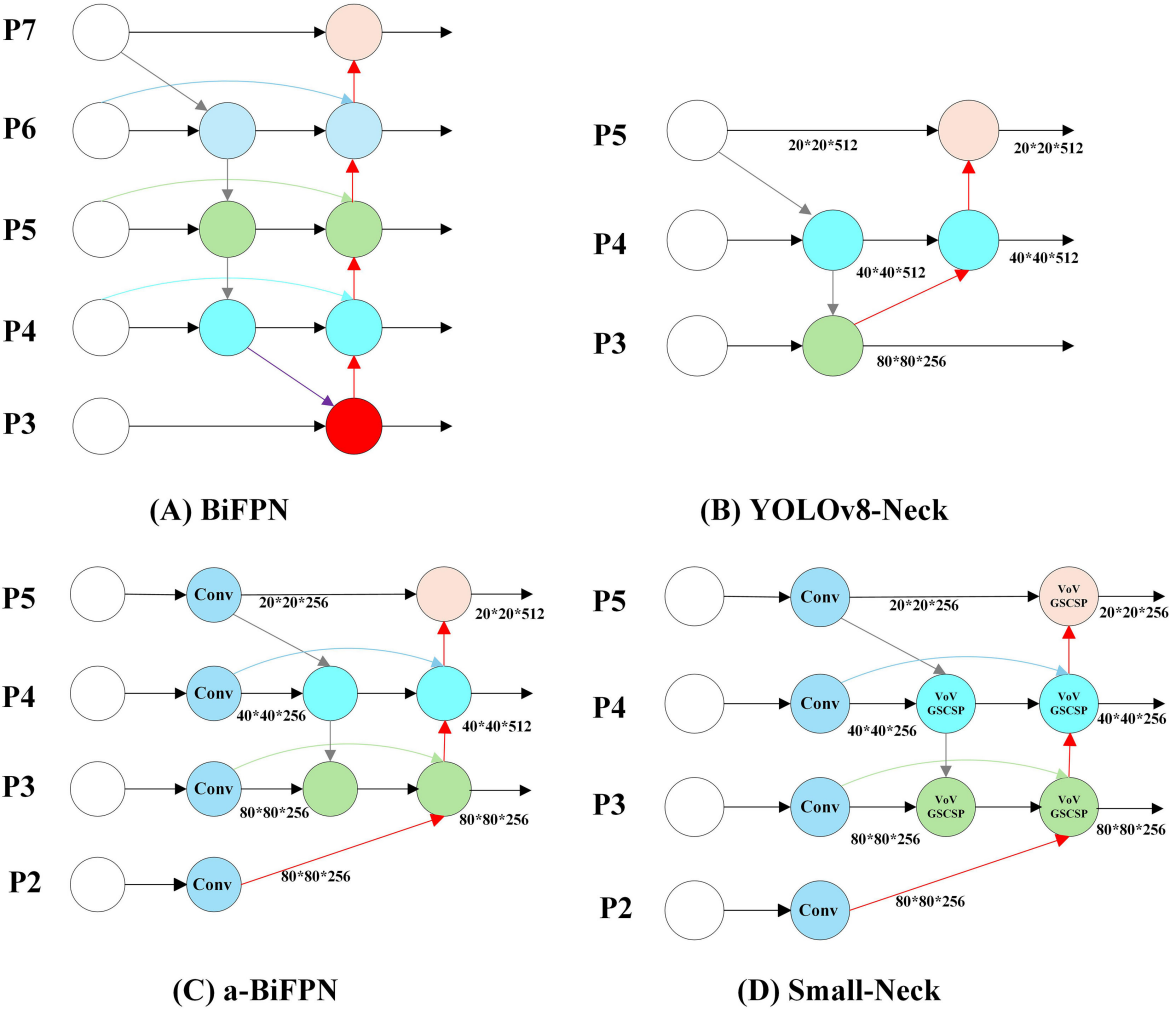
Small-neck feature fusion network architecture. **(A)** BiFPN: achieves efficient bidirectional feature fusion for multi-scale information flow. **(B)** Original YOLOv8 feature fusion network: adopts a PANet-style structure to enable both top-down and bottom-up feature aggregation. **(C)** a-BiFPN: introduces an additional P2 shallow feature layer, which preserves finer spatial details crucial for small object detection and enhances small-target perception. **(D)** Small-Neck: builds upon the a-BiFPN design, incorporating the more efficient GSConv convolution module and VoVGSCSP fusion block. Furthermore, channel pruning is applied based on the characteristics of the Pest24 dataset, achieving a balance between lightweight and efficient network design.

In YOLOv8, the feature fusion network adopts a PAN-FPN ( (Liu et al., 2018) (Lin et al., 2017) structure similar to that of YOLOv5 (Jocher et al., 2020), as shown in **Figure 5B**. This design aims to enhance the model’s ability to detect objects at different scales through top-down and bottom-up feature fusion. However, this feature fusion network faces several challenges in small pest detection tasks. Multiple downsampling operations in the YOLOv8 neck cause the loss of shallow features that are essential for identifying small pests. Moreover, the fusion process focuses mainly on high-level semantic features, resulting in insufficient utilization of shallow spatial details.

To improve the representation of small-scale features, an enhanced feature fusion module named a-BiFPN (**Figure 5C**) is designed based on the YOLOv8 neck. Unlike the original PAN-FPN structure, a-BiFPN introduces an additional P2 shallow feature layer, which contains finer spatial information critical for small-object detection. The fusion of P2–P5 layers is realized through bidirectional top-down and bottom-up pathways similar to BiFPN(**Figure 5A**) (Tan et al., 2020), enabling effective multi-level feature integration. Experimental results on the Pest24 dataset demonstrate that this modification significantly improves the detection accuracy of small pests.

**Figure 6:**
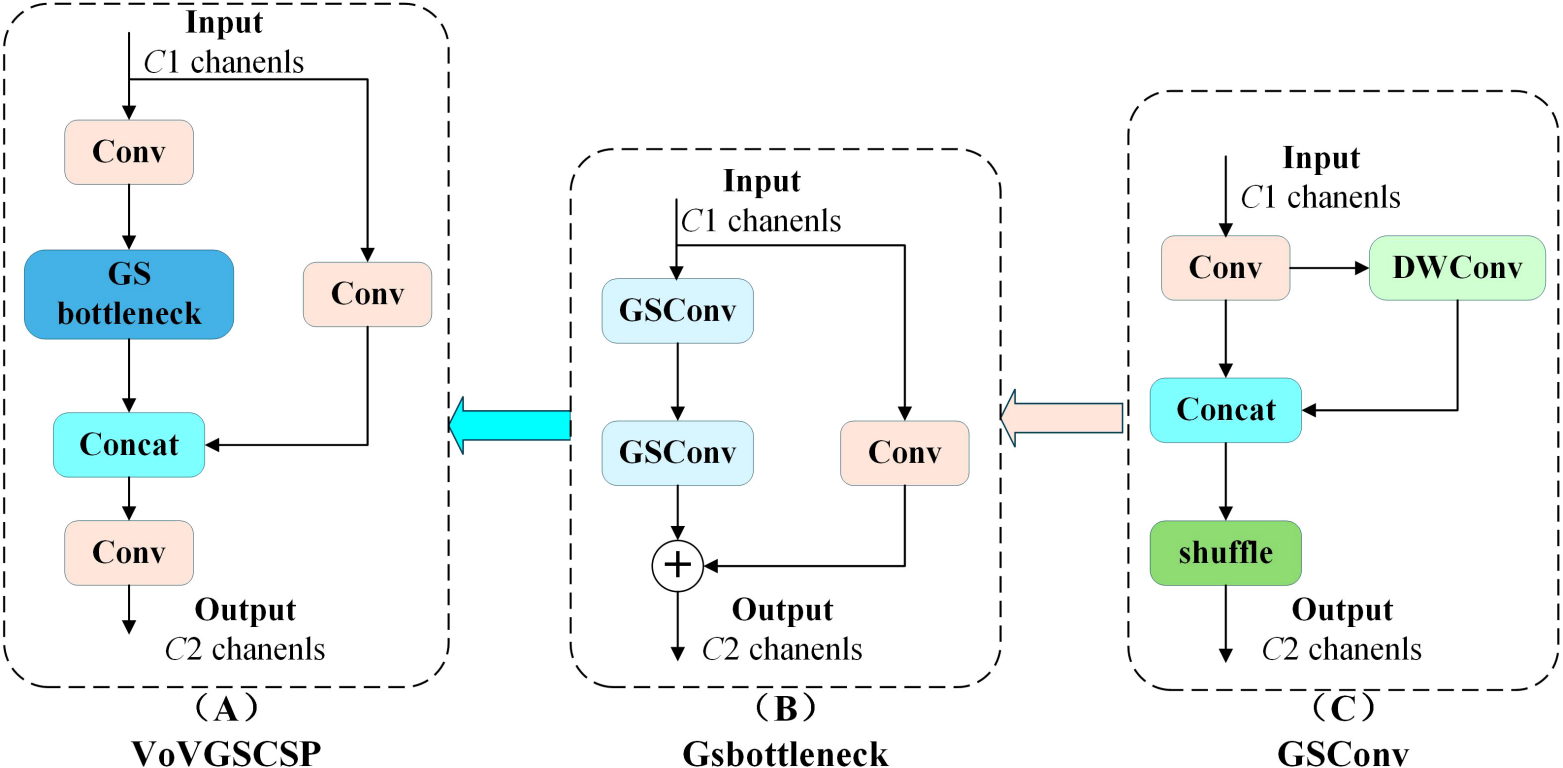
GSConv and VoVGSCSP module structure.

Although a-BiFPN enhances small-object detection performance, its fusion of deep-level features (P4 and P5) introduces redundant computation for the Pest24 dataset, which mainly consists of small pest targets. Considering that the contribution of these deep-level features is relatively limited, the proposed Small-Neck **Figure 5D**) simplifies the a-BiFPN by applying a dataset-specific channel pruning strategy. Specifically, the output channels of the P3–P5 layers are uniformly reduced to 256 to minimize unnecessary computation while retaining essential shallow and mid-level features for accurate small-object detection. In addition, standard convolutions are replaced by the more efficient GSConv module (**Figure 6C**) to accelerate inference and enhance real-time performance, while the original C2f module is substituted with VoVGSCSP (**Figure 6A**) (Li et al., 2022b) to improve feature reuse and gradient propagation. As a result, Small-Neck achieves a favorable balance among detection accuracy, computational complexity, and inference speed.

Considering that input features generated from different layers vary in importance and require an efficient and stable feature fusion strategy, this paper adopts the Fast Normalized Fusion method proposed by Tan et al. (2020). This method optimizes the weight normalization approach to ensure model training stability while accelerating convergence, thereby improving the effectiveness of feature fusion. Taking the fusion of two features at the P4 layer in Small-Neck as an example:

(7) P4td=Conv(w1·P4in+w2·Resize(P5in)w1+w2+ξ)

(8) $$\begin{array}{c} P_{4}^{out}=Conv\left(\frac{w_{1}\prime \cdot {\text{P}}_{4}^{\text{in}}+{\text{w}}_{2}\prime \cdot P_{4}^{td}+w_{3}\prime \cdot \text{Resize}\left(P_{4}^{out}\right)}{w_{1}\prime +w_{2}\prime +w_{3}\prime +\text{ξ}}\right) \end{array}$$

In Equations 7, 8, P4td represents the intermediate feature at the P4 layer in the top-down path; P4out represents the output feature at the P4 layer in the bottom-up path. The Resize operation refers to either downsampling or upsampling. The parameter w is learned during training and is used to measure and differentiate the importance of different feature maps in the fusion process for the final output.

#### 2.2.4 Inner-WIoU loss function

**Figure 7:**
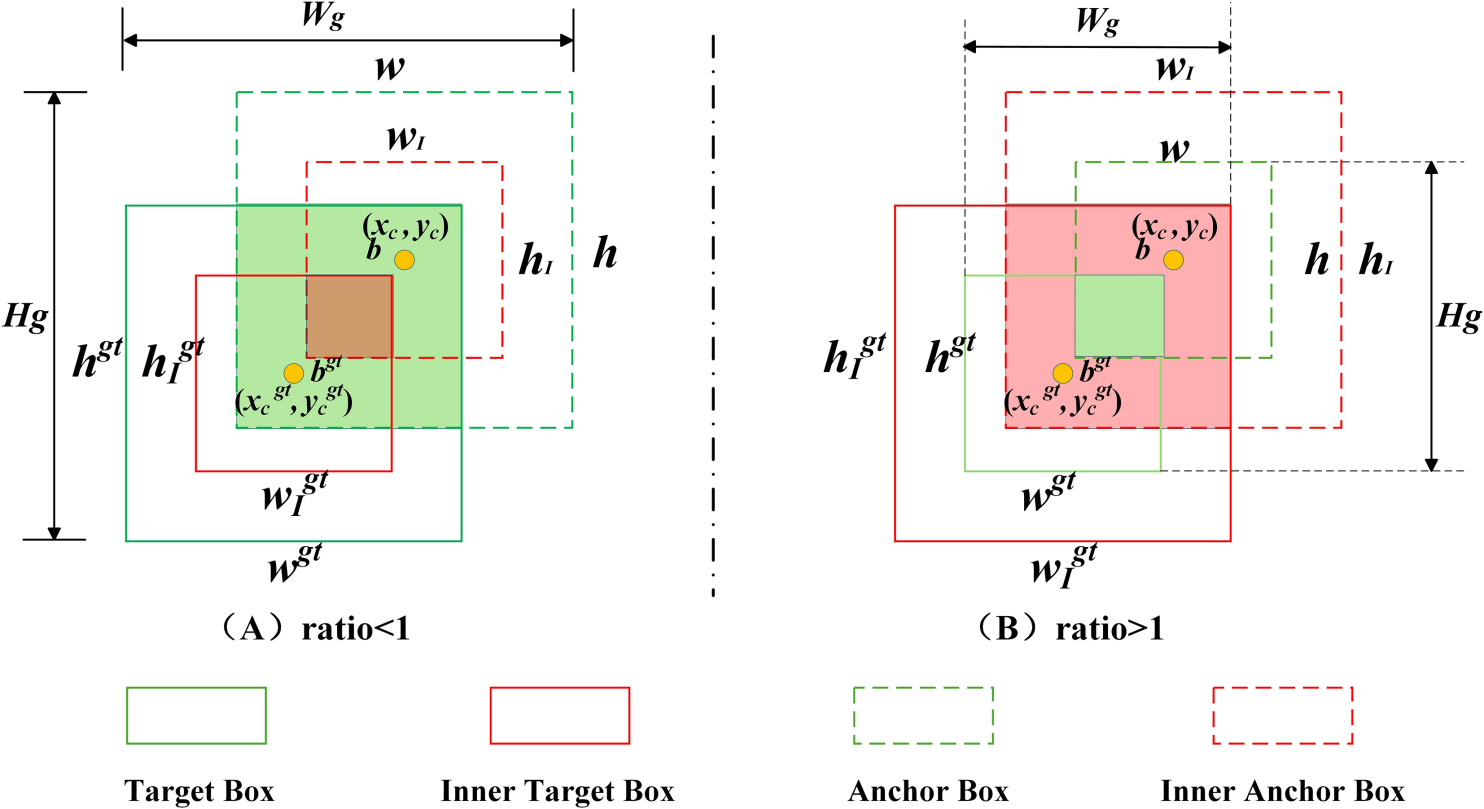
Inner-IoU mechanism diagram.

The YOLOv8 model adopts the CIoU (Zheng et al., 2020) loss function for bounding box regression, aiming to optimize the regression by jointly considering the distance between box centers, aspect ratio, and overlap area. However, CIoU lacks adaptive adjustment capability across different detection tasks, resulting in limited generalization. To accelerate bounding box regression for small pests and enhance the model’s performance in small pest detection. This paper introduces a scale factor from Inner-IoU (Zhang et al., 2023) into the WIoU (Tong et al., 2023) loss function to control the auxiliary boxes, enabling the model to focus more on small pest regions. Therefore, the Inner-WIoU loss function is proposed in this paper, and its mechanism is illustrated in **Figure 7**.

In bounding box regression tasks, IoU (Yu et al., 2016) loss accurately describes the matching degree between the predicted box and the ground truth (GT) box, ensuring that the model effectively learns object localization information. As shown in Equation 9, B and Bgt represent the predicted box and the ground truth box, respectively.

(9) $$\begin{array}{c} IoU=\frac{|B\;\cap B^{gt}|}{|B\;\cup B^{gt}|} \end{array}$$

The WIoUv3 loss function introduces a focusing mechanism by constructing a method for computing gradient gain, and it proposes a reasonable strategy for gradient gain allocation. This strategy reduces the competitiveness of high-quality anchor boxes and also mitigates the harmful gradients produced by low-quality anchor boxes. Therefore, WIoUv3 can effectively focus on hard samples anchor boxes, helping the model pay more attention to these challenging samples during training, thereby improving its generalization ability. Specifically, WIoUv3 assigns differentiated gradient gains to different anchor boxes during gradient computation. This ensures that the network focuses more on hard samples while reducing the excessive influence of easy samples on model training. High-quality anchor boxes typically have high IoU with ground truth (GT) boxes and are easier to predict accurately. Therefore, the WIoUv3 loss reduces their influence to prevent them from dominating the training process. For low-quality anchor boxes, WIoUv3 decreases their gradient contributions to avoid negative effects on model training. Through this gradient gain allocation strategy, WIoUv3 significantly improves the model’s performance in detecting small pests and handling dense regions, thereby enhancing its generalization ability.

(10) $$\begin{array}{c} L_{IoU}=1-IoU \end{array}$$

(11) $$\begin{array}{c} R_{WIoU}=exp\left(\frac{{\left(x_{c}-x_{c}^{gt}\right)}^{2}+{\left(y_{c}-y_{c}^{gt}\right)}^{2}}{{\left(W_{g}+H_{g}\right)}^{2}}\right) \end{array}$$

(12) $$\begin{array}{c} L_{WIoUv1}=R_{WIoU}L_{IOU} \end{array}$$

(13) $$\begin{array}{c} L_{WIoUv3}=rL_{WIoUv1},r=\frac{\text{β}}{{\text{δα}}^{\text{β}-\text{δ}}},\beta =\frac{L_{IoU}^{*}}{\bar{{\text{L}}_{\text{IoU}}}}\in \left[0,+\infty \right) \end{array}$$

In Equations 10–13, *R_WIoU_* denotes the exponential transformation of the normalized length between the center points. Wg and Hg represent the width and height of the union region between the predicted box and the ground-truth box. $L_{IoU}^{*}$ is the monotonic focusing factor, $\bar{L_{IoU}}$ is the momentum-based moving average, and β is the non-monotonic focusing factor.

In this study, inspired by Inner-IoU, a scaling factor named ratio is introduced into the WIoUv3 loss to control the auxiliary bounding box. This allows the model to focus more on small pest regions. Based on the original WIoUv3 loss, the proposed Inner-WIoU further enhances attention to small pests, ensuring that the auxiliary box contributes effectively to regression accuracy while reducing unnecessary computational cost. The detailed computation process is illustrated in **Figure 7**, and the mathematical formulation of the loss function and auxiliary box regression is provided in Equations 14–20.

(14) $$\begin{array}{c} b_{\text{l}}^{gt}=x_{c}^{gt}-\frac{w^{gt}\cdot ratio}{2},b_{r}^{gt}=x_{c}^{gt}+\frac{w^{gt}\cdot ratio}{2} \end{array}$$

(15) $$\begin{array}{c} b_{t}^{gt}=y_{c}^{gt}-\frac{h^{gt}\cdot ratio}{2},b_{b}^{gt}=y_{c}^{gt}+\frac{h^{gt}\cdot ratio}{2} \end{array}$$

(16) $$\begin{array}{c} b_{l}=x_{c}-\frac{w\cdot ratio}{2},b_{r}=x_{c}+\frac{w\cdot ratio}{2} \end{array}$$

(17) $$\begin{array}{c} b_{t}=y_{c}-\frac{h\cdot ratio}{2},b_{b}=y_{c}+\frac{h\cdot ratio}{2} \end{array}$$

(18) $$\begin{array}{c} \text{inter=}\left(\text{min}\left(b_{r}^{gt},b_{r}\right)-\text{max}\left(b_{l}^{gt},b_{l}\right)\right)\cdot \left(\text{min}\left(b_{b}^{gt},b_{b}\right)-\text{max}\left(b_{t}^{gt},b_{t}\right)\right) \end{array}$$

(19) $$\begin{array}{c} union=\left(w^{gt}\cdot h^{gt}\right)\cdot {\left(ratio\right)}^{2}+\left(w\cdot h\right)\cdot {\left(ratio\right)}^{2}-\text{inter} \end{array}$$

(20) $$\begin{array}{c} L_{Inner-WIoU}=L_{WIoUv3}+IoU-\frac{\text{inter}}{union} \end{array}$$

In Equations 14–20, $\left(x_{c}^{gt},y_{c}^{gt}\right)$ are the center coordinates of the Target Box and the Inner Target Box. $\left(x_{c},y_{c}\right)$ are the center coordinates of the Anchor Box and the Inner Anchor Box. hgt and wgt are the height and width of the target box. h and w are the height and width of the anchor box. ratio∈[0.5,1.5] is the scaling factor; $L_{Inner-WIoU}$ is the final Inner-WIoU loss function.

Based on the study by Zhang et al., the default value of the ratio is set to 0.7 (Zhang et al., 2023). When the ratio is less than 1 (as shown in **Figure 7A**), the scale of the auxiliary bounding box is smaller than that of the actual bounding box, resulting in a regression effective range smaller than that of the IoU loss. At this time, the absolute gradient of the auxiliary bounding box is greater than that of the IoU loss, accelerating the convergence of high-IoU samples. When the ratio is greater than 1 (as shown in **Figure 7B**), the larger-scale auxiliary bounding box expands the effective range of regression, providing gains for low-IoU regressions. Since the dataset used in this study contains a large number of small pests, a more detailed investigation of the ratio value was conducted. Specifically, experiments were performed within the range of [1, 1.2], with an interval of 0.05. The results show that when the ratio is set to 1.05, the model achieves the best performance in terms of mean Average Precision (mAP), as well as the highest detection accuracy for small pests. Further analysis reveals that, at this value, the regression range of the auxiliary bounding box is well-balanced. It enables better capture of small-object features while avoiding excessive focus on large targets, thereby improving detection performance for small pests.

## 3 Results

### 3.1 Experimental setup

The experiments were conducted using the PyTorch 1.11.0 framework. The detailed environment configurations are listed in **Table 1**. For training settings, the batch size was set to 32, with a total of 200 epochs. The initial learning rate was 0.01, weight decay was set to 0.0005, and the momentum parameter was set to 0.937. All models were trained from scratch without using any pretrained weights. Stochastic Gradient Descent (SGD) was adopted as the optimizer to improve the model’s ability to escape local optima. To enhance the training performance of the model, a combination of the Cosine Annealing learning rate scheduler and the Early Stopping mechanism was employed. The Cosine Annealing strategy dynamically adjusts the learning rate during training, gradually reducing it in later stages to facilitate stable convergence near the optimal solution and avoid local minima. The Early Stopping mechanism monitors the validation loss, and if no significant decrease is observed over 50 consecutive epochs, training is automatically terminated to prevent overfitting and reduce training time. The synergy between these two strategies effectively optimizes the training process and improves the model’s generalization performance on the validation set.

**Table 1:** Hardware and software environment.

Configuration Item	Training device	Edge computing device
CPU	Intel(R) Xeon(R) Gold 5318S CPU @ 2.10GHz	Raspberry Pi 4B
GPU	NVIDIA A100(80GB)	–
CUDA	11.3	–
Deep learning frame	Pytorch 1.11.0	Pytorch 1.11.0
Programming Language	Python 3.9.7	Python 3.9.7
Operating System	Ubuntu 20.04.5 LTS	Ubuntu server 24.04

### 3.2 Evaluation metrics

In pest detection tasks, it is essential to balance detection accuracy and model complexity. To comprehensively evaluate the detection performance of the proposed model, this study adopts Precision, Recall, and mean Average Precision (mAP) as the primary evaluation metrics. Meanwhile, to measure model complexity, we report the number of parameters and Giga Floating Point Operations per Second (GFLOPs). In addition, Frames Per Second (FPS) is used to assess the real-time inference performance of the model. The calculation methods of these metrics are provided in Equations 21–26:

(21) $$\begin{array}{c} \mathrm{Pr}ecision=\frac{TP}{TP+FP}\times 100\% \end{array}$$

(22) Recall=TPTP+FN×100%

In Equations 21, 22: TP(True Positive): the number of pest samples correctly detected by the model. FN(False Negative): the number of actual pest samples that were not detected by the model. TN(True Negative): TN(True Negative): the number of non-pest samples correctly identified as non-pests by the model. FP(False Positive): the number of non-pest samples incorrectly detected as pests by the model.

(23) $$\begin{array}{c} m\text{AP}=\frac{1}{n}\sum_{1}^{n}P_{i}=\frac{1}{n}\left(P_{1}+P_{2}+\cdots +P_{n}\right) \end{array}$$

(24) $$\begin{array}{c} mAP@50\sim 95=\frac{1}{c}\sum_{k=1}^{c}mAP@50_{k} \end{array}$$

(25) $$\begin{array}{c} mAP@50\sim 95=\frac{1}{10}\left(mAP@50+mAP@55+\cdots mAP95\right) \end{array}$$

(26) $$\begin{array}{c} FPS=\frac{1}{T} \end{array}$$

In Equations 23–26: mAP@0.5 represents the mean Average Precision when the Intersection over Union(IoU) threshold is set to 0.5. mAP@0.5~0.95 refers to the average of mAP values calculated at multiple IoU thresholds ranging from 0.5 to 0.95 with a step size of 0.05. Compared to mAP@0.5, this metric is more stringent and provides a more comprehensive evaluation of the model’s detection performance. T denotes the processing time required for a single input image. FPS measures the number of images the model can process per second during inference, reflecting its real-time performance.

### 3.3 Result and analysis

#### 3.3.1 Training and validation of the YOLO-DCPG algorithm

**Figure 8:**
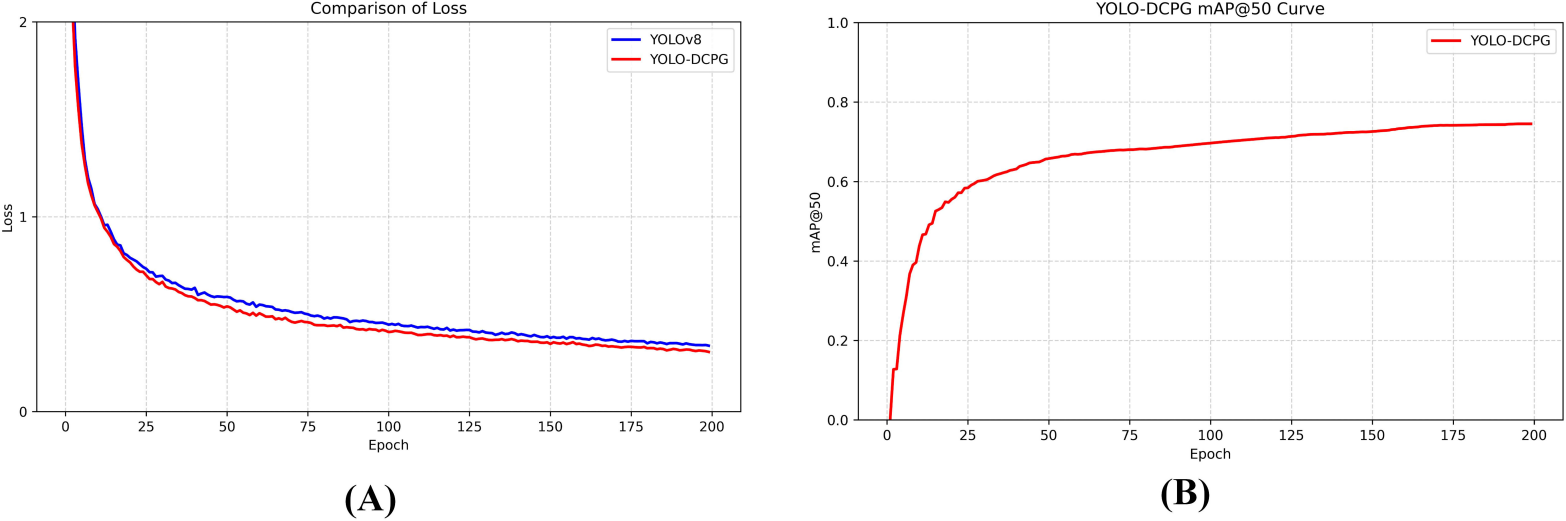
The training loss curve variation **(A)** and mAP@50 training variation **(B)**.

**Figure 8A** illustrates the comparison of training loss curves between the baseline YOLOv8 model and the proposed YOLO-DCPG model. At the early stages of training, both models exhibit a rapid decline in training loss, indicating efficient feature learning. YOLO-DCPG demonstrates a noticeably faster convergence rate compared to the baseline, reflecting the overall effectiveness of the proposed improvements. As training progresses, the loss curve of YOLO-DCPG stabilizes earlier and maintains lower fluctuations, suggesting better training stability and stronger generalization capability, which confirms the effectiveness of the overall network optimization strategy.

In **Figure 8B**, the variation of mAP@50 across epochs illustrates the model’s performance evolution during training. The mAP@50 rises sharply in the early phase, indicating a rapid improvement in detection accuracy. As training proceeds, the curve gradually levels off, and by around the 160th epoch, the model’s performance approaches its optimal state. Combined with **Figure 7A**, it can be observed that the proposed YOLO-DCPG not only converges faster but also exhibits better generalization capability, verifying the overall effectiveness of the improvements.

#### 3.3.2 Comparison with state-of-the-art methods

To evaluate the effectiveness of the YOLO-DCPG model proposed in this paper for pest detection tasks, its performance is compared with several mainstream object detection models, including YOLOv3-tiny (Adarsh et al., 2020), YOLOv5n (Jocher et al., 2020), YOLOv6n (Li et al., 2022a), YOLOv8n (Varghese and Sambath, 2024), YOLOv10n (Wang et al., 2024), YOLOv11n (Khanam and Hussain, 2024), YOLOv12n (Tian et al., 2025) and NanoDet-Plus-m-1.5x (Lyu, 2020) as well as Transformer-based (Lv et al., 2024) models such as RT-DETR-n (Zhao et al., 2024) and RT-DETR-resnet18 (Qin et al., 2024). The comparison results are shown in **Table 2**.

**Table 2:** The performance comparison of different object detection models.

Model	Precision (%)	mAP@50 (%)	mAP@50~95 (%)	GFLOPs (G)	Parameters (M)	FPS (Image/s)	Model Size (M)
YOLOv3-tiny	76.9	74.3	48.0	14.5	9.54	121.9	18.3
YOLOv5n	78.7	72.5	45.7	5.7	2.01	92.6	4.1
YOLOv6n	75.3	68.3	42.5	11.6	4.16	101.0	8.2
YOLOv10n	74.9	73.0	46.7	8.4	2.72	90.9	5.5
YOLOv11n	73.0	72.8	46.6	6.5	2.59	84.0	5.2
YOLOv12n	79.5	71.5	45.1	6.5	2.57	102.0	5.3
NanoDet-Plus-m-1.5x	70.3	67.3	40.2	2.97	2.44	117.6	4.7
RT-DETR-n	81.2	74.5	46.1	37.9	15.57	34.1	30.0
RT-DETR-resnet18	77.8	71.5	40.4	58.6	20.2	48.3	42.0
YOLOv8n	75.6	73.2	46.6	8.2	3.02	105.3	6.0
YOLO-DCPG(our)	80.1	74.0	47.5	5.7	1.47	98.0	3.2

In comparison with lightweight YOLO series object detection models, the YOLO-DCPG model proposed in this paper demonstrates strong overall performance. With a model size of 3.2 MB and a computational cost of 5.7 GFLOPs, It is the most resource-efficient models among all YOLO variants. Although YOLOv3-tiny achieves an mAP@50 that is 0.5% higher than YOLO-DCPG, YOLO-DCPG delivers comparable detection performance with only one-fifth of the resource consumption. In addition, YOLO-DCPG achieves a detection precision of 80.1%, which is higher than YOLOv3-tiny’s 76.9%. Although YOLOv5 and YOLOv12 achieve higher inference speeds, YOLO-DCPG demonstrates superior detection performance, with improvements of 1.5% and 2.5% in mAP@50, and 1.8% and 2.4% in mAP@50–95, respectively. YOLOv6n reaches 68.3% for mAP@50 and 42.5% for mAP@50~95, both clearly lower than YOLO-DCPG. Its computational cost is 11.6 GFLOPs, almost twice that of YOLO-DCPG, and it has 4.16M parameters, which means higher resource usage. YOLOv10n and YOLOv11n achieve mAP@50~95 scores of 46.7% and 46.6%, respectively, which are slightly lower than that of YOLO-DCPG. Their inference speeds are also slower. In addition, both models have larger sizes and higher computational complexity compared to YOLO-DCPG. Therefore, YOLO-DCPG achieves a better balance among accuracy, computational cost, and inference efficiency.

Compared with NanoDet-Plus-m-1.5x, the proposed YOLO-DCPG achieves a 6.7% improvement in mAP@50 and a 7.3% improvement in mAP@50~95. Meanwhile, its inference speed reaches 98 FPS, slightly lower than 117.6 FPS of NanoDet-Plus-m-1.5x.These results demonstrate that YOLO-DCPG achieves a better balance between detection performance and inference speed, offering superior overall efficiency. Compared with the Transformer-based RT-DETR models, YOLO-DCPG also shows clear advantages in lightweight design. RT-DETR-n has 15.57M parameters, 37.9 GFLOPs of computation, and a model size of 30 MB. In contrast, YOLO-DCPG has only 1.47M parameters, 5.7 GFLOPs, and a model size of 3.2 MB. These are about one-tenth of RT-DETR-n. In terms of accuracy, YOLO-DCPG is slightly lower than RT-DETR-n in mAP@50. However, it performs better in mAP@50~95. RT-DETR-resnet18 has even higher computational complexity. But its accuracy is not better. Its mAP@50~95 is only 40.4%. YOLO-DCPG reaches an inference speed of 98 FPS. RT-DETR-n and RT-DETR-resnet18 run at only 34.1 FPS and 48.3 FPS. This shows that YOLO-DCPG is more efficient for edge device deployment.

Compared with the baseline model YOLOv8n, YOLO-DCPG shows better performance with only a slight drop in inference speed. Precision, mAP@50, and mAP@50~95 increase by 4.6%, 0.8%, and 0.9%, respectively. At the same time, the model size, GFLOPs, and number of parameters are reduced by 46.7%, 30.1%, and 51.2%, respectively. This improvement mainly comes from the DCPGAttention module. It helps the model focus more on pest target regions and improves feature discrimination in complex backgrounds. This increases the ability to distinguish between different classes and improves detection accuracy. The model also uses the lightweight StarNet-S100 as the backbone for feature extraction, and a Small-Neck structure for feature fusion. These components help make better use of shallow features, which improves the detection of small pests. Experimental results show that YOLO-DCPG reduces model complexity while maintaining high detection accuracy. It achieves a good balance between precision and lightweight design.

#### 3.3.3 Ablation study

To evaluate the performance of YOLO-DCPG in pest detection tasks, ablation experiments were conducted on the Pest24 dataset. The tested components include StarNet-S100, Small-Neck, DCPGAttention, and Inner-WIoU. The results are shown in **Table 3**. Compared with the baseline model YOLOv8n, the improved YOLO-DCPG model reduces the number of parameters by 51.2% and lowers the computational cost (GFLOPs) by 46.7%. At the same time, detection performance improves. Precision, mAP@50, and mAP@50~95 increase by 4.5%, 0.8%, and 0.9%, respectively. These results show that the improvements in model structure and loss function help reduce model complexity while maintaining detection performance.

**Table 3:** Ablation experiment results.

Model	Precision (%)	Recall (%)	mAP@50 (%)	mAP@50~95 (%)	GFLOPs (G)	Parameters (M)
YOLOv8n	75.6	67.4	73.2	46.6	8.2	3.02
YOLOv8n+DCPGAttention	78.5	69.9	75.0	48.7	8.2	3.02
YOLOv8n+StarNet	80.0	67.4	72.9	46.2	6.9	2.39
YOLOv8n+Small-Neck	79.9	69.4	74.3	47.6	6.6	1.88
YOLOv8n+StarNet+Small-Neck	77.5	68.9	73.5	46.9	5.7	1.47
YOLOv8n+StarNet+Small-Neck+ DCPGAttention	78.1	69.6	73.7	47.5	5.7	1.47
YOLOv8n+StarNet+Small-Neck+ DCPGAttention+Inner-WIoU (YOLO-DCPG)	80.1	69.2	74.0	47.5	5.7	1.47

For the feature extraction network, this paper replaces the original YOLOv8n backbone with the StarNet-S100 network. Compared with the original YOLOv8n backbone, StarNet-S100 shows a slight drop in mAP but has a clear advantage in parameter size. In terms of attention mechanism, this paper adds the DCPGAttention module to the bottom of the YOLOv8n backbone. This module combines the control gate from GCT with dual pooling on the mean and standard deviation of the input features. It helps the model capture global information more effectively. After adding this module, the model reaches a Precision of 78.5%, mAP@50 of 75.0%, and mAP@50~95 of 48.7%. These are improvements of 2.9%, 1.8%, and 2.1% over the original YOLOv8n. For feature fusion, this paper simplifies the Neck part of YOLOv8n by replacing it with a lightweight Small-Neck. Compared with YOLOv8n, Small-Neck reduces the number of parameters by 37.7% and GFLOPs by 19.5%. At the same time, it improves performance in Precision, Recall, mAP@50, and mAP@50~95. For the loss function, this paper introduces a scale factor into the WIoU loss. This factor adjusts the size of the auxiliary box. It helps the model focus more on small pest regions and improves the detection of small objects.

To evaluate the combined effect of each module, this paper designs an ablation study with step-by-step integration of the four proposed components. The results are shown in **Table 3**. The combination of StarNet-S100 and Small-Neck reduces parameters by 51.2% and GFLOPs by 30.1%, while slightly improving accuracy. After adding the DCPGAttention module, mAP@50 increases by 0.2% and mAP@50~95 by 0.6%. Finally, by integrating all modules, the complete YOLO-DCPG model is formed. Without significantly increasing computation, it further improves Precision by 2.0% and mAP@50 by 0.3% compared to the previous version. These results confirm the effectiveness of the combined improvements and their synergy.

#### 3.3.4 Comparison of different lightweight backbone networks

For backbone selection, this paper conducts comparative experiments on StarNet-S50 (Ma et al., 2024), StarNet-S100 (Ma et al., 2024), StarNet-S150 (Ma et al., 2024), MobileNetv4-small (Qin et al., 2024), and FasterNet-t0 (Chen et al., 2023). The goal is to evaluate the performance of different lightweight networks on the pest detection task. The focus is on their impact on model parameters, GFLOPs, FPS, and detection accuracy. The results are shown in **Table 4**. In terms of detection accuracy, FasterNet-t0 achieved the best performance, with a Precision of 80.6% and an mAP@50 of 73.1%, indicating strong detection capability. However, it has significantly higher GFLOPs and parameter count compared to other networks, and its inference speed is slower. These factors limit its suitability for deployment on edge devices. MobileNetv4-small shows lower detection accuracy and higher computational complexity in the pest detection task, making it less suitable for efficient deployment scenarios. StarNet-S50 has the smallest number of parameters and the fastest inference speed, but its accuracy drops noticeably. StarNet-S150 achieves similar accuracy to StarNet-S100 but requires more computation. In contrast, StarNet-S100 achieves a good balance between performance and efficiency. It maintains low computation (6.9 GFLOPs) and a low parameters(2.39M), while reaching high detection accuracy. Its inference speed reaches 106.4 FPS, making it a well-balanced choice for both accuracy and speed.

**Table 4:** Comparison of different lightweight backbones.

Model	Precision (%)	Recall (%)	mAP@50 (%)	mAP@50~95 (%)	GFLOPs (G)	Parameters (M)	FPS (Image/s)
YOLOv8n	76.9	66.3	71.3	45.0	8.2	3.02	105.3
+StarNet-S50	78.0	66.4	71.0	44.3	5.3	1.89	111.1
+StarNet-S100	80.0	67.4	72.9	46.2	6.9	2.39	106.4
+StarNet-S150	79.0	68.4	72.9	46.6	7.6	2.90	104.2
+MobileNetv4-small	76.0	64.3	68.9	42.9	21.4	5.38	90.1
+FasterNet-t0	80.6	68.3	73.1	46.3	10.7	4.18	92.6

#### 3.3.5 Comparison of attention modules

To verify the effectiveness of the proposed DCPGAttention module in pest detection tasks, this paper uses YOLOv8n as the baseline model. Five attention mechanisms are tested: GCT (Yang et al., 2020), SRM (Lee et al., 2019), SE (Hu et al., 2018), ECA (Wang et al., 2020b), and the proposed DCPGAttention. All other components are kept unchanged to ensure fair comparison.

The experimental results are shown in **Table 5**. All attention modules improve the detection performance to varying degrees, although they slightly increase GFLOPs and parameters. SE achieves the best Recall and FPS, but its mAP@50 and mAP@50~95 are slightly lower than those of DCPGAttention. GCT and SRM show advantages in Precision and Recall, but their overall mAP scores are relatively low. ECA achieves a similar inference speed to DCPGAttention, but still lags in mAP. Overall, DCPGAttention achieves the best mAP performance, with an mAP@50 of 75.0% and an mAP@50~95 of 48.7%. This suggests that the combination of dual channel pooling and gate control mechanisms effectively captures global features and improves detection performance. Meanwhile, DCPGAttention maintains a comparable parameter and computational cost to other attention modules, and reaches an inference speed of 114.9 FPS, showing good efficiency and real-time capability.

**Table 5:** Comparison of different attention models’ performance.

Model	Precision (%)	Recall (%)	mAP@50 (%)	mAP@50~95 (%)	GFLOPs (G)	Parameters (M)	FPS (Image/s)
YOLOv8n	75.5	67.4	73.2	46.6	8.2	3.02	105.3
+GCT	78.8	70.0	74.3	48.1	8.2	3.02	113.6
+ECA	78.5	69.5	74.4	48.3	8.2	3.02	114.9
+SRM	79.0	70.0	74.0	47.8	8.2	3.02	114.9
+SE	78.2	70.1	74.5	48.5	8.2	3.03	116.3
+DCPGAttention	78.5	69.9	75.0	48.7	8.2	3.02	114.9

**Figure 9:**
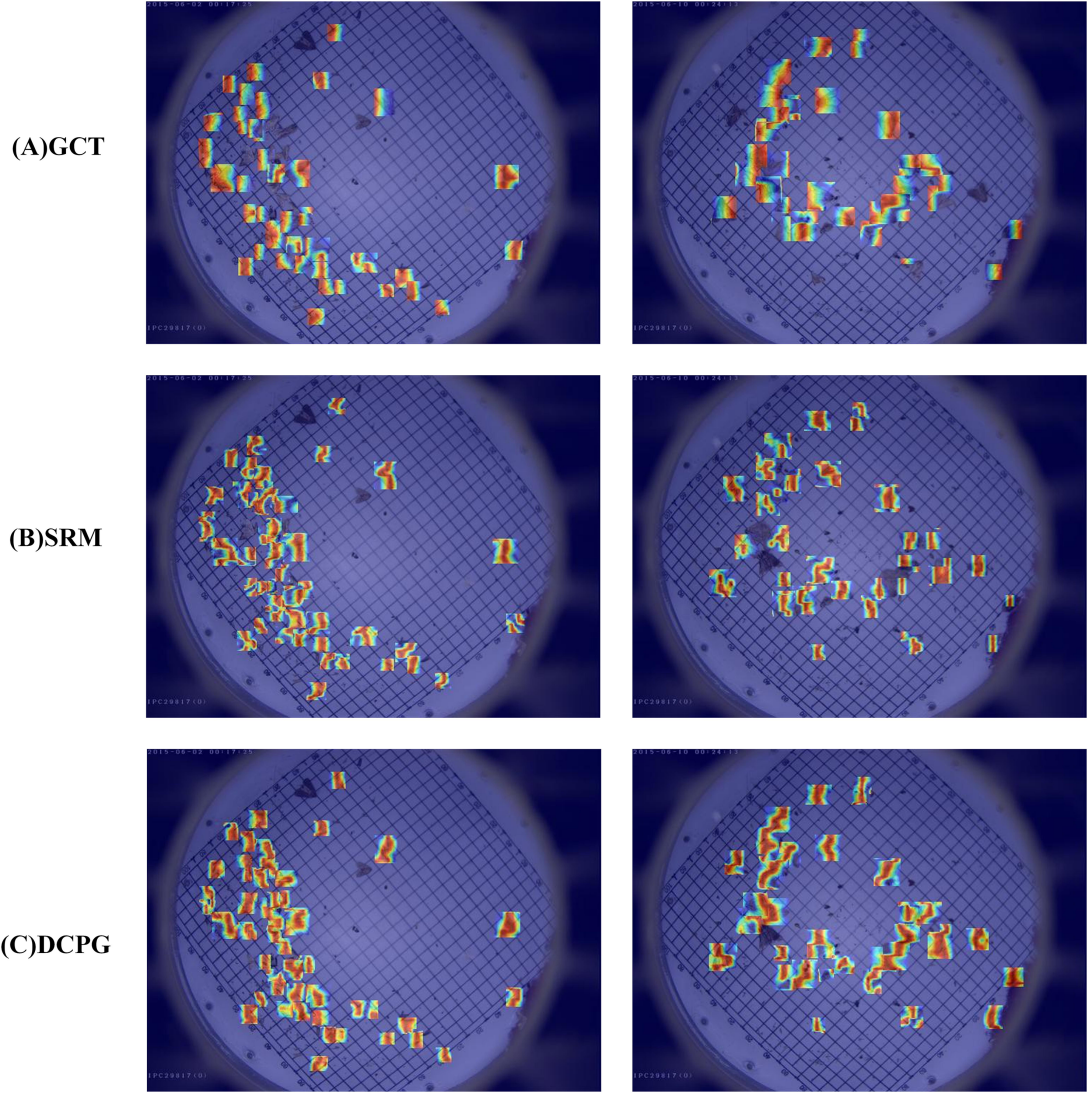
Visualization of attention heatmap. Where warmer colors represent higher attention intensity, highlighting the regions most focused by the model during pest detection.

To further illustrate the influence of the DCPGAttention mechanism on feature learning, attention heatmaps were generated for the highly relevant GCT, SRM, and DCPGAttention modules for comparison. As shown in **Figure 9**, different attention modules guide the model to focus on distinct spatial regions, revealing their varying abilities to capture critical target features. Specifically, the GCT module tends to produce attention over a relatively large area, but some targets are missed and the focus is dispersed, lacking concentration on key regions. The SRM module improves upon this by concentrating attention more effectively, but the focused regions are still limited, and some pests are not effectively captured by the attention mechanism. The DCPGAttention module, while occasionally missing individual pests, demonstrates a more concentrated and comprehensive focus compared to GCT and SRM, effectively capturing both the target body and edges. This indicates that DCPGAttention provides improved attention distribution, highlighting critical features necessary for accurate pest detection more effectively than the other two modules.

#### 3.3.6 Small-neck and channel pruning

Different feature fusion networks have a key impact on the model’s detection performance. To verify the advantage of the improved lightweight Small-Neck in small pest detection tasks, ablation experiments were conducted based on the YOLOv8n baseline. The results are shown in **Table 6**. After introducing the enhanced a-BiFPN, the model’s detection precision improved from 75.5% to 80.0%, and mAP@50 increased to 74.4%, with almost no impact on inference speed. Then, integrating GSConv and VoVGSCSP structures further reduced computation and parameters, while maintaining stable accuracy. However, inference speed slightly decreased. Finally, considering that pests in the Pest24 dataset are generally small, we observed redundancy in the feature fusion layers P4 and P5 designed for large objects. Therefore, we pruned the channel numbers of P4 and P5 to 256, forming the final Small-Neck structure. Although Small-Neck caused a slight drop in accuracy, it significantly reduced parameters and computation. Its FPS increased to 108.7, greatly improving deployment efficiency on edge devices and meeting practical application requirements.

**Table 6:** Comparison of different attention models’ performance.

Model	Precision (%)	Recall (%)	mAP@50 (%)	mAP@50~95 (%)	GFLOPs (G)	Parameters (M)	FPS (Image/s)
YOLOv8n	75.5	67.4	73.2	46.6	8.2	3.02	105.3
+a-BiFPN	80.0	70.0	74.4	48.0	7.9	2.85	105.3
+GSConv+VoVGSCSP	76.0	69.7	74.2	47.8	7.4	2.81	104.2
+a-BiFPN+GSConv+VoVGSCSP	80.1	69.9	74.6	47.5	7.0	2.65	101.1
Small-Neck(Channel Pruning)	79.9	69.4	74.3	47.6	6.6	1.88	108.7

#### 3.3.7 Comparison of different IoU loss functions

To evaluate the impact of different regression loss functions on detection performance, this paper uses YOLO-DCPG as the baseline model. The tested losses include CIoU, SIoU (Gevorgyan, 2022), DIoU (Zheng et al., 2020), WIoU v1 (Tong et al., 2023), WIoU v2 (Tong et al., 2023), WIoU v3 (Tong et al., 2023), and Inner-WIoU. All experiments are conducted under the same training settings and dataset. The comparison focuses on differences in localization accuracy and pest detection ability. The results are shown in **Table 7**. Compared with CIoU, SIoU, DIoU, WIoU v1, WIoU v2, and WIoU v3 improve detection precision but show varying degrees of decline in Recall. Among them, WIoU v3 achieves the best mAP@50 performance.

**Table 7:** Comparison of different loss functions.

IoU	Precision (%)	Recall (%)	mAP@50 (%)	mAP@50~95 (%)	GFLOPs (G)	Parameters (M)
CIoU	78.1	69.6	73.5	46.9	5.7	1.47
SIoU	78.8	69.0	73.5	47.4	5.7	1.47
DIoU	79.4	69.2	73.6	47.4	5.7	1.47
WIoU v1	79.6	69.0	73.1	47.2	5.7	1.47
WIoU v2	78.3	69.1	73.4	47.3	5.7	1.47
WIoU v3	80.1	69.1	73.8	47.2	5.7	1.47
Inner-WIoU(radio=0.7)	81.0	67.9	73.4	47.2	5.7	1.47
Inner-WIoU(radio=1.2)	74.9	70.0	73.7	47.8	5.7	1.47
Inner-WIoU(radio=1.15)	80.5	69.1	73.6	47.6	5.7	1.47
Inner-WIoU(radio=1.1)	76.4	69.3	73.6	48.0	5.7	1.47
Inner-WIoU(radio=1.05)	80.1	69.2	74.0	47.5	5.7	1.47

Based on this, we introduce a scale factor called ratio into the WIoU v3 loss function, using Inner-IoU to control the size of the auxiliary box. This helps the model focus more on small pest regions and effectively improves bounding box regression accuracy. When the ratio is set to 0.7, the model achieves the highest Precision, but the Recall drops significantly. To further optimize this parameter, we conducted a systematic experiment with a step size of 0.05 in the range [1, 1.2]. The results show that setting the ratio to 1.05 leads to the best mAP@50 improvement. It also provides a better balance across multiple metrics, including Precision, Recall, and mAP@50~95, resulting in more stable and effective supervision during training.

#### 3.3.8 Visualization of model predictions

**Figure 10:**
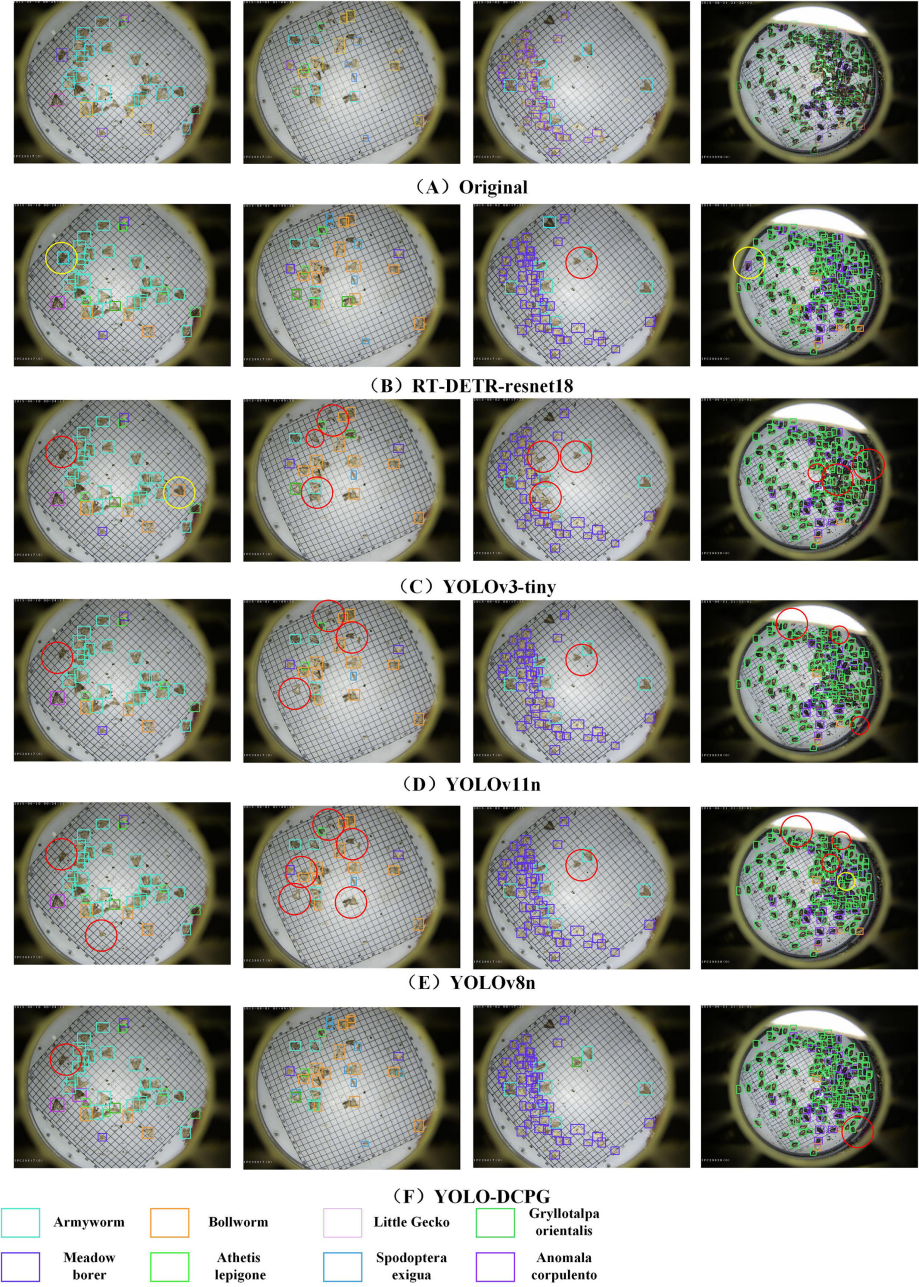
Visualization results of different models. The red circles indicate missed detections, while the yellow circles indicate false detections.

To provide a more comprehensive comparison of detection performance across models, this paper presents visualization results of Original, RT-DETR-resnet18 (Zhao et al., 2024), YOLOv3-tiny (Adarsh et al., 2020), YOLOv11n (Khanam and Hussain, 2024), YOLOv8n (Varghese and Sambath, 2024), and the proposed YOLO-DCPG. The results are shown in **Figure 10**. From left to right, the number of pests in the sample images gradually increases, making the detection task more challenging. For images with fewer pests, most models perform reasonably well. RT-DETR-resnet18, which has the largest number of parameters, shows the best performance in these cases. However, as pest density increases, performance differences between models become more noticeable. YOLOv11n struggles in high-density scenes, with many missed detections. YOLOv3-tiny and YOLOv8n also show some missed and overlapping boxes when detecting small pests or dense clusters. In contrast, YOLO-DCPG, with structural improvements over YOLOv8n, significantly reduces model parameters and computational cost while maintaining high detection accuracy. Even in high-density, multi-target scenarios, YOLO-DCPG demonstrates strong robustness and generalization capability.

#### 3.3.9 Generalization evaluation on other datasets

To evaluate the generalization capability of the YOLO-DCPG module, experiments were conducted on the RP11 dataset using both the baseline YOLOv8n and the proposed YOLO-DCPG model. RP11 is a real-world dataset containing 4,559 images of 11 rice pest species. The dataset was divided into training, validation, and test sets in a 7:2:1 ratio, resulting in 3,191 training images, 912 validation images, and 456 test images. The detection results of the two models on this dataset are shown in **Table 8**.

**Table 8:** Comparison of different attention models’ performance.

Model	Precision (%)	mAP@50 (%)	mAP@75 (%)	mAP@50~95 (%)	GFLOPs (G)	Parameters (M)
YOLOv8n	89.2	85.4	79.2	70.2	8.2	3.02
YOLO-DCPG(our)	90.3	87.8	82.8	71.3	5.7	1.47

**Figure 11:**
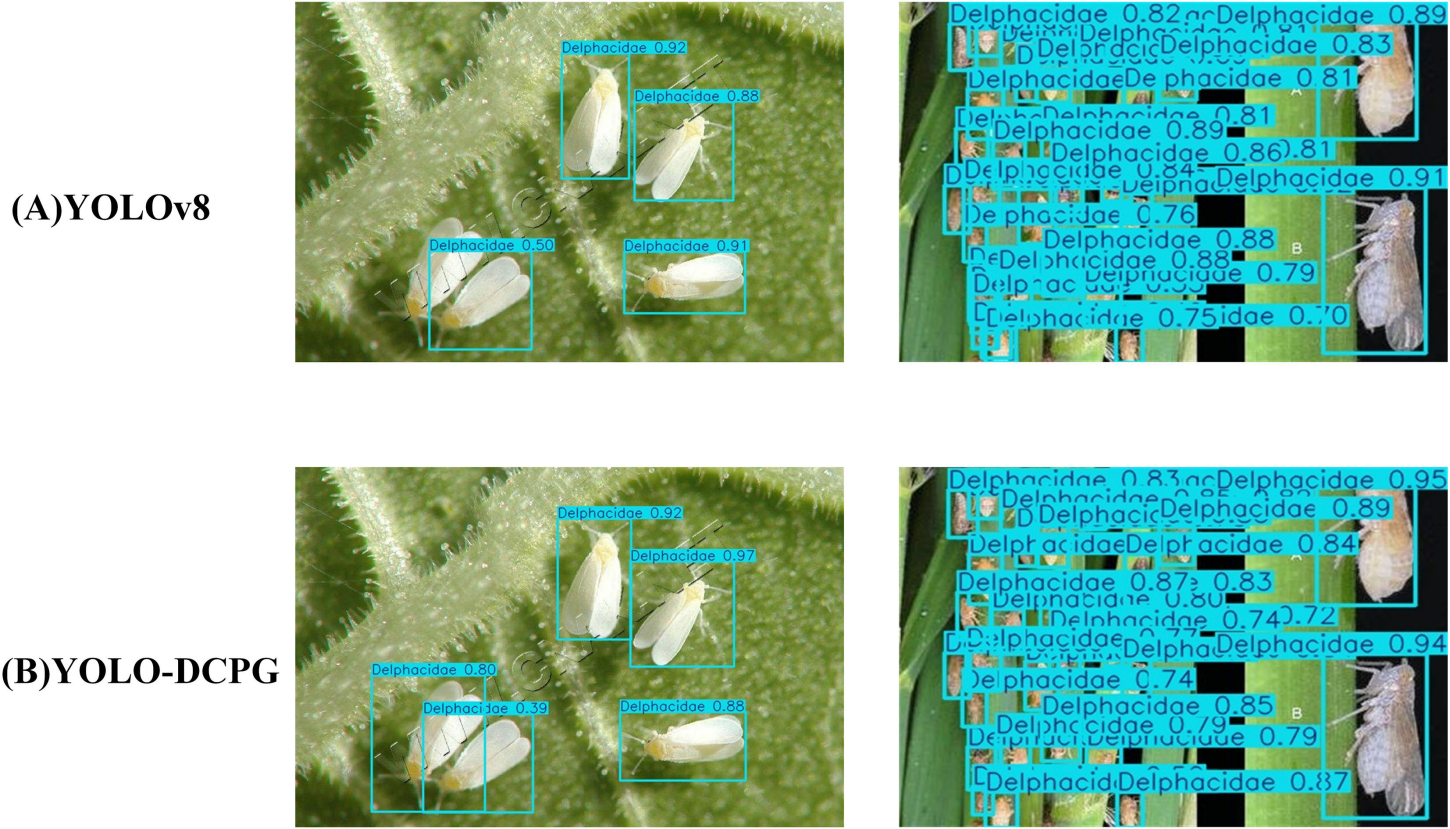
Visualization results of different models.

As shown in **Table 8**, the YOLO-DCPG model achieves a precision of 90.3%, mAP@50 of 87.8%, mAP@75 of 82.8%, and mAP@50~95 of 71.3% on the RP11 dataset. Compared with the baseline YOLOv8n, these correspond to improvements of 1.1%, 2.4%, 3.6%, and 1.1%, respectively. In addition, YOLO-DCPG significantly reduces computational cost and model size, with GFLOPs decreasing from 8.2 to 5.7 and parameters reduced from 3.02M to 1.47M. The results demonstrate that YOLO-DCPG enhances detection performance and efficiency, confirming its effectiveness and broad applicability. To provide a clearer comparison of the models’ detection performance, the visualization results are presented in **Figure 11**.

### 3.4 Edge deployment and visualization results

#### 3.4.1 Deployment on edge devices

**Figure 12:**
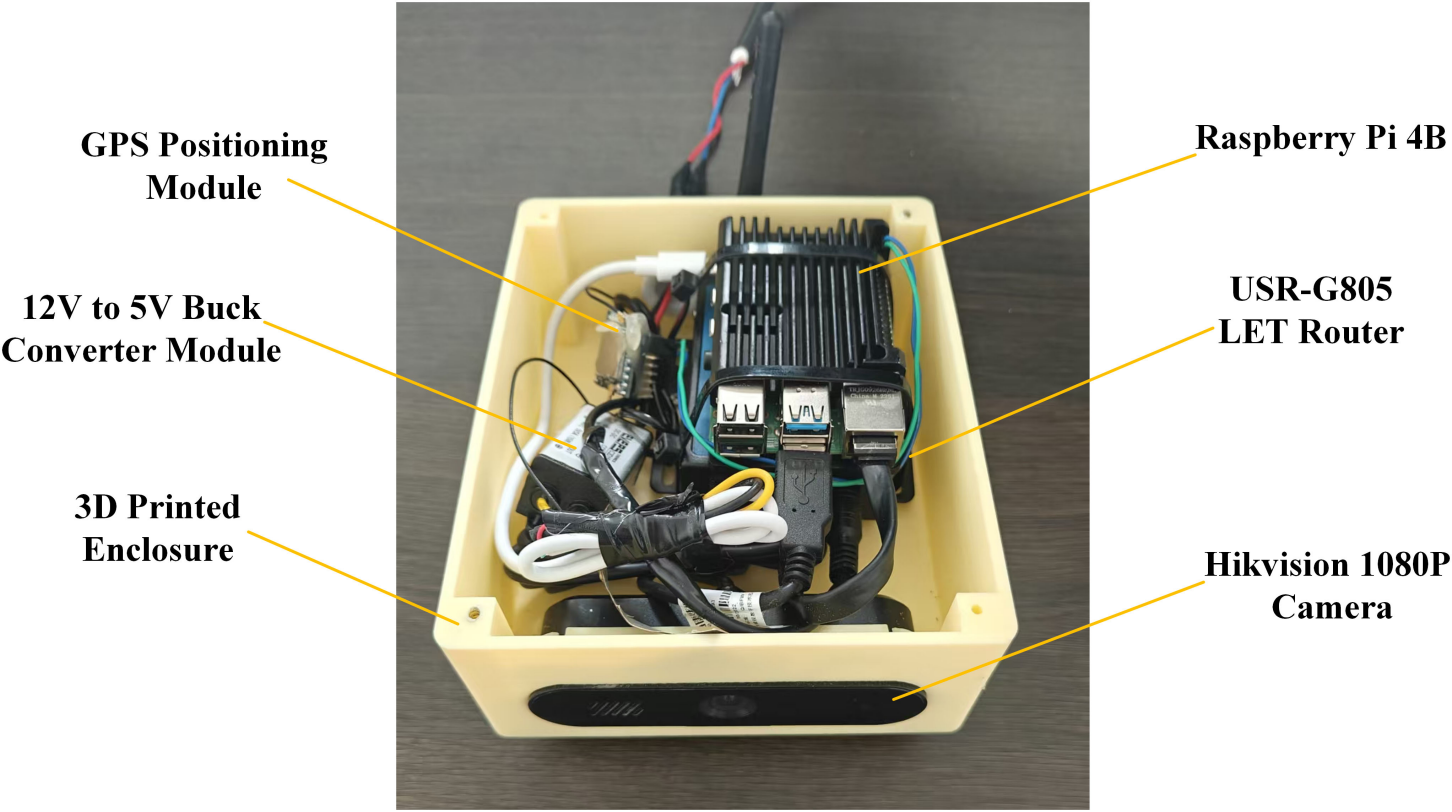
Photo of the device.

To verify the practical applicability of the proposed model in real agricultural environments, this study deploys the model on an edge computing device based on the Raspberry Pi platform. Specifically, a Raspberry Pi 4B is used as the core hardware. It is equipped with a Hikvision camera, a 4G network module, a voltage regulation circuit, and a custom 3D-printed case to ensure system stability and environmental adaptability. The assembled device is shown in **Figure 12**.

To meet the limited computational resources of edge devices, this paper adopts the lightweight backbone StarNet-S100 during model training. In addition, a Small-Neck feature fusion module combined with channel pruning is designed to effectively reduce the model’s parameters and computational complexity. During deployment, the trained model is converted to the ONNX format and integrated into the Raspberry Pi environment. ONNX Runtime is used as the inference engine. It is optimized for ARM architecture and supports multi-threading, as well as fast loading and execution of lightweight models. This setup enables the YOLO-DCPG model to achieve a real-time inference speed of 12.4 FPS on the Raspberry Pi 4B. It ensures efficient and stable operation on edge devices.

#### 3.4.2 Visualization of inference results on edge computing device

**Figure 13:**
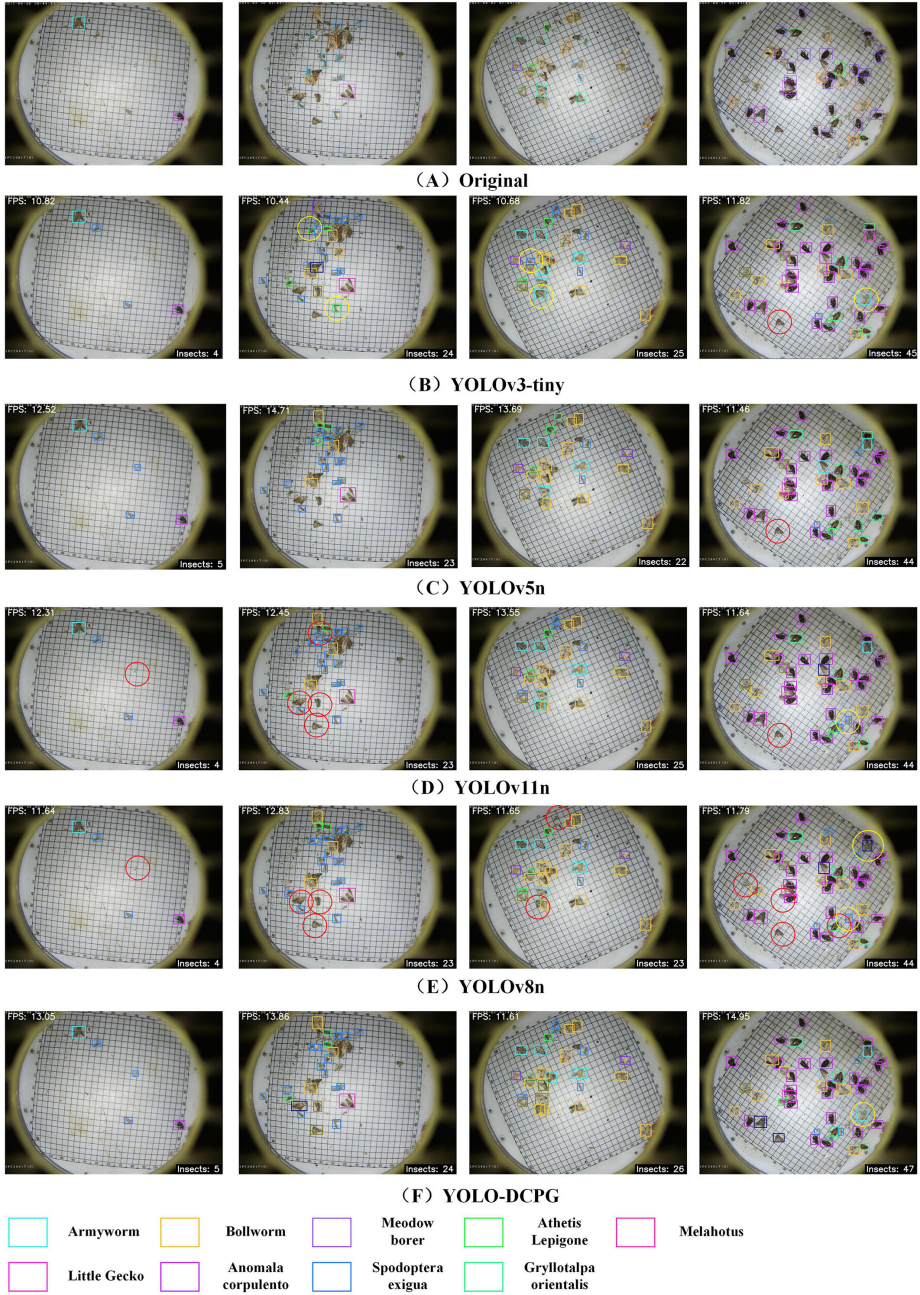
Visualization of inference results on raspberry Pi. The red circles indicate missed detections, while the yellow circles indicate false detections.

To evaluate the detection performance of models on edge devices, this paper compares five lightweight models: YOLOv3-tiny (Adarsh et al., 2020), YOLOv5n (Jocher et al., 2020), YOLOv11n (Khanam and Hussain, 2024), YOLOv8n (Varghese and Sambath, 2024), and YOLO-DCPG. All models were exported to the ONNX format and deployed on a Raspberry Pi 4B for inference testing. The results are shown in **Figure 13**. The top-left corner of each image displays the current inference frame rate (FPS), while the bottom-right corner shows the number of detected pests. To further compare inference speed and detection performance, **Table 9** summarizes the number of detected pests, average FPS, and ONNX model size for each model.

**Table 9:** Inference speed and detection performance of different pest detection models on the Raspberry Pi platform.

Model	Number of detected pests	FPS (Image/s)	ONNX model size (M)
YOLOv3-tiny	98	11.0	36.3
YOLOv5n	94	13.1	7.7
YOLOv11n	96	12.5	9.9
YOLOv8n	94	12.0	10.0
YOLO-DCPG(our)	102	13.4	5.5

As shown in **Table 9** and **Figure 13**, all models perform well when detecting simple sample images. However, as the number of pests increases, YOLO-DCPG detects 102 pest samples, while YOLOv5n and YOLOv8n detect only 94, indicating noticeable missed detections. YOLOv3-tiny and YOLOv11n achieve similar results but still fail to identify some targets. In terms of inference speed, YOLO-DCPG reaches 13.4 FPS on the Raspberry Pi, exceeding YOLOv3-tiny, YOLOv5n, YOLOv11n, and YOLOv8n by 2.4, 0.3, 0.9, and 1.4 FPS, respectively. Moreover, its ONNX model size is only 5.5 MB, the smallest among all compared models. YOLO-DCPG provides comparable inference speed and higher detection accuracy on the Raspberry Pi, identifying more pest targets and better satisfying the dual requirements of real-time performance and accuracy in agricultural applications. Therefore, YOLO-DCPG demonstrates higher practical value for edge device deployment.

## 4 Discussion

To balance high precision pest detection and edge deployment efficiency, this paper proposes a pest detection model based on dual channel pooling gated attention. This attention module uses both average pooling and standard deviation pooling channels to effectively enhance the model’s focus on key pest regions. In the feature extraction stage, the lightweight backbone StarNet-S100 is used. It significantly reduces the model size while maintaining good feature representation. This facilitates deployment on edge devices. In the feature fusion stage, a Small-Neck structure is designed. It combines an improved a-BiFPN and GSConv modules to achieve efficient multi-scale feature fusion and transmission. To improve small object detection, the Inner-WIoU loss function is introduced in the regression loss. The scale factor ratio guides the model to better focus on tiny pests, enhancing localization accuracy and speeding up convergence. On the Pest24 tiny pest dataset, YOLO-DCPG achieves a Precision of 80.1%, mAP@50 of 74.0%, and mAP@50~95 of 47.5%. The model’s computation is 5.7 GFLOPs with only 1.47 million parameters, and the final model size is limited to 3.2 MB. Compared to the baseline YOLOv8n, the parameters, computation, and model size are reduced by 51.2%, 30.1%, and 46.7%, respectively. This significantly improves the model’s suitability for deployment on edge devices. It provides a more efficient and practical solution for agricultural pest monitoring in limited-resource environments.

Although this study has made progress in tiny pest detection, some limitations remain. On one hand, the model still suffers from missed and false detections in complex scenes with high object density or occlusion. On the other hand, the current dataset has limited coverage and cannot fully represent the diversity of pests in real field environments. To improve the model’s generalization and practical performance, future work will include continuous collection of real-world images, expansion of the dataset, and incremental annotation and training. In addition, the model will be migrated to high-performance edge devices such as Jetson Nano to further enhance system responsiveness and real-time performance, better meeting the needs of agricultural pest monitoring in real-world applications.

## Data Availability

The original contributions presented in the study are included in the article/supplementary material. Further inquiries can be directed to the corresponding authors.
